# High-resolution gridded soil moisture and soil temperature datasets for the Indian monsoon region

**DOI:** 10.1038/sdata.2018.264

**Published:** 2018-11-20

**Authors:** H. P. Nayak, K. K. Osuri, Palash Sinha, Raghu Nadimpalli, U. C. Mohanty, Fei Chen, M. Rajeevan, D. Niyogi

**Affiliations:** 1 School of Earth Ocean and Climate Sciences, Indian Institute of Technology, Bhubaneswar, Odisha – 752050, India; 2 Centre for Oceans, Rivers, Atmosphere and Land Sciences, Indian Institute of Technology Kharagpur- 721302, India; 3 Department of Agronomy and Department of Earth, Atmospheric, and Planetary Sciences, Purdue University, West Lafayette, IN47907, USA; 4 Department of Earth and Atmospheric Sciences, National Institute of Technology, Rourkela, Odisha – 769 008, India; 5 National Center of Atmospheric Research, Boulder, Colorado 80301, USA; 6 State Key Laboratory of Severe Weather, Chinese Academy of Meteorological Science, Beijing, China; 7 Ministry of Earth Sciences, Prithvi Bhavan, Lodhi Road, New Delhi- 110003, India

**Keywords:** Hydrology, Water resources, Geography, Hydrology

## Abstract

High-resolution soil moisture/temperature (SM/ST) are critical components of the growing demand for fine-scale products over the Indian monsoon region (IMR) which has diverse land-surface characteristics. This demand is fueled by findings that improved representation of land-state help improve rainfall/flood prediction. Here we report on the development of a high-resolution (4 km and 3 hourly) SM/ST product for 2001–2014 during Indian monsoon seasons (June–September). First, the quality of atmospheric fields from five reanalysis sources was examined to identify realistic forcing to a land data assimilation system (LDAS). The evaluation of developed SM/ST against observations highlighted the importance of quality forcing fields. There is a significant relation between the forcing error and the errors in the SM/ST. A combination of forcing fields was used to develop 14-years of SM/ST data. This dataset captured inter-annual, intra-seasonal, and diurnal variations under different monsoon conditions. When the mesoscale model was initialized using the SM/ST data, improved simulations of heavy rain events was evident, demonstrating the value of the data over IMR.

## Background & Summary

The IMR is characterized by diverse land surface characteristics (topography, vegetation, soil) and rainfall distribution. Surface heterogeneity has a positive feedback on precipitation intensity^1^. The IMR has also been identified as a hot spot^2,3^ for soil moisture (SM) - rainfall coupling. Earlier studies have demonstrated the significance of land surface on weather and climate^4–10^, agriculture^11^, and hydrology^12^. The SM state is being extensively used to improve water and agriculture models/practices^13–15^.

Land surface observations, however, are generally available only for select locations. This constraint the representation of spatiotemporal surface variability and the associated predictability of weather and climate models^16^. Further, the representability of the SM/ST measurements is limited and a dense network is hard to operate beyond a watershed. One way to address this need of locally representative but regionally variable SM/ST fields is to create high-resolution surface conditions using a land data assimilation system (LDAS^17^), and is the focus of this study.

In recent years, there has been success in preparing regional SM/ST fields using LDAS^10,18,19^. A LDAS uses observed/analyzed land parameters (vegetation, landuse/landcover, soil characteristics) and atmospheric fields such as temperature (T2) and specific humidity at 2 m (Q2), surface winds at 10 m height, surface pressure (PSFC), rainrate, and surface downward shortwave (SWRAD) and longwave (LWRAD) radiation^17^. Though the global reanalysis products provide an attractive data source, they have a relatively poor resolution (e.g., GLDAS is typically available at 100 km grid spacing and a 25 km product is also recently available). The reliability of these datasets needs to be evaluated. The IMR, in particular, has lagged in addressing the evaluation of the land-surface forcing fields and its impact on simulated land surface products.

Accordingly, this study seeks to- (i) evaluate the land surface forcing products, (ii) assess the relation between quality of forcing and offline prepared SM/ST, (iii) analyze land surface features in association with the Indian summer monsoon, and (iv) demonstrate the value of the SM/ST data taking example of numerical weather prediction of heavy rain events. The ultimate goal is to develop a gridded SM/ST data for the monsoon community.

It is postulated that the quality of the forcing to LDAS will impact the resulting SM/ST fields. Data from Modern Era Retrospective-analysis for Research and Applications (MERRA^20^), MERRA Version-2 (MERRA2^21^), National Centers for Environmental Prediction – Climate Forecast System Reanalysis (NCEP-CFSR^22^), European Centre for Medium-Range Weather Forecasts Re-Analysis(ERA-Interim^23^), and Global Land Data Assimilation System (GLDAS^19^) reanalysis are tested using in-situ measurements (automatic weather stations (AWS), Agromet stations). Statistical analyses indicate that T2 and PSFC from CFSR and GLDAS radiation (SWRAD, LWRAD) can be the better-input forcing for developing LDAS fields over India (refer section ‘Validation of atmospheric forcing’). Numerical integrations are also conducted using the reanalysis datasets at 4 km spatial resolution for 2009–2013. Among the input parameters, Tropical Rainfall Measuring Mission (TRMM^24^) rainrate, Indian satellite-derived landuse^25^, United State Geological Survey soil texture^26^, and green vegetation fraction^27^ are specified identically for each of the analyses. Integrating the LDAS with the ‘better-input’ forcing led to SM/ST fields with better skill in reproducing the observations. The SM/ST fields were then developed for 14 years (2001–2014) using the said forcing input. The developed fields were validated for 2011–2013 due to the limited availability of observations over the IMR. This is both the limitation of the study, and the motivation for creating such a dataset.

This newly developed high spatiotemporal resolution (4 km, 3 hourly) SM/ST dataset for 2001–2014 over the IMR can be used for various hydro-meteorological applications. The utilization of these SM/ST in initializing Weather Research and Forecasting^28^ (ARW) model improved the simulation of heavy rainfall associated with monsoon depressions and convective events. Long-term LDAS products replicated the diurnal variation, and the seasonal and the inter-annual variability of SM/ST. However, the diurnal variation of ST over complex terrain remains challenging. Interestingly, the Himalayan regions are well captured, likely due to the utilization of Indian landuse/cover data in the LDAS. The inter-annual variations of SM are also better captured in the dataset than GLDAS when compared with satellite merged product. The new datasets are also expected to be of value to studies that need a verification/comparison of SM/ST fields over the data sparse IMR.

## Methods

### Work flow for generation of the data

The preparation of SM/ST data follows four major steps. (i) The first step is to evaluate the quality of forcing inputs to the LDAS over the IMR. The evaluation of forcing parameters is carried out for five sets of data sources as given in Table 1. (ii) The second step is to integrate the LDAS at 4 km grid spacing for the period of 2009–2013 over the IMR with input from all the forcings. The 4 km SM/ST products are being developed as they are considered useful for severe weather prediction, hydrological studies, and agriculture over the IMR with diverse surface characteristics which demand relatively high resolution regional land information. The 4 km spacing is considered optimal in terms of the availability of relatively high-fidelity rainfall fields. The SM/ST derived from LDAS (and GLDAS) products is validated against the Indian in-situ observations. (iii) The land surface conditions are developed for 14 years (2001–2014) considering the combination of input forcing and the output from LDAS is compared with station observations. (iv) The value of the data product is demonstrated with utilization of these SM/ST in initializing ARW model fields. The details of land data assimilation, data sources used, and numerical experiments conducted are presented next.

### Land data assimilation system

The community version of LDAS^17^ (version 3.4.1) available through the National Center for Atmospheric Research (NCAR) is used to generate SM/ST fields in the present study. The version used includes a glacial ice and sea ice module. It considers four soil layers at 0–10 cm, 10–40 cm, 40–100 cm, and 100–200 cm with a total soil depth of 2 m. The vegetation root depth varies with land use type in the upper 1.5 m of soil. The LDAS is designed for both uncoupled and coupled modes within the ARW^28^. The advantage is that the LDAS uses the same grid as the Weather Research Forecasting suite of models, sharing the same Noah^29^ land model and same geophysical parameters (land use, soil texture, terrain height, time-varying vegetation fields) and reduces interpolation errors that affect the coupled model integration. The Noah LSM integrates hydro-meteorological forcing parameters, and static surface fields; and estimates SM, ST, evapo-transpiration, fluxes, interception, and other land variables (complete list available from^17^). The Noah-LSM is based on the Penman potential evaporation approach^30^, the multi-layer soil model^31^, and a primitive simple canopy model^32^. It is further improved with the addition of complex canopy resistance^33,34^ and frozen ground physics^35^.

### Reanalysis products and observations

The near-surface forcing products with varying spatiotemporal resolution are obtained from MERRA^20^, MERRA2^21^, NCEP-CFSR^22^, ERA-Interim^23^, and GLDAS^19^. Brief synopsis on spatio temporal resolution for each of the reanalysis products are given in Table 1. The validation of the reanalysis products is carried out at station points using an inverse distance weighted interpolation^36^ technique and the validation is confined for 2011–2013 considering the availability of observations. The TRMM provides 3 hourly gridded rainfall estimates over the tropical region and has been evaluated^37,38^ over Indian region. The TRMM-3B42 could capture high rainfall over Western Ghats and Himalayan foothills during monsoon season and in that regards is considered better than the Global Precipitation Climatology Project (GPCP) rainfall^37^. Further, the TRMM fields could detect large daily events reasonably well, but had lower skill in specifying moderate and light event amounts on short time intervals^39^. The rainfall product is comparatively closer to the ground-truth and has better performance over the west coast of India even in comparison with the Climate Prediction Center Morphing (CMORPH), Naval Research Laboratory (NRL) –blended, Precipitation Estimation from Remotely Sensed Information using Artificial Neural Networks (PERSIANN) rainfall product at daily scale^38^. For the TRMM and other datasets there are known issues regarding rainfall detection over the rain-shadow region of southeast peninsular India, semi-arid north-west parts of India, and hilly northern parts. Prior study^38^ also suggest that TRMM-TMPA merged rainfall product can be used for monsoon studies over Indian land region. In view of above studies, the gridded 3 hourly TRMM rainfall analysis (mm hr^−1^) available at 0.25° × 0.25° resolution is interpolated linearly to the hourly rainrate. Note that the rain gauge observations are not included in this rainfall analyses.

**Figure 1:**
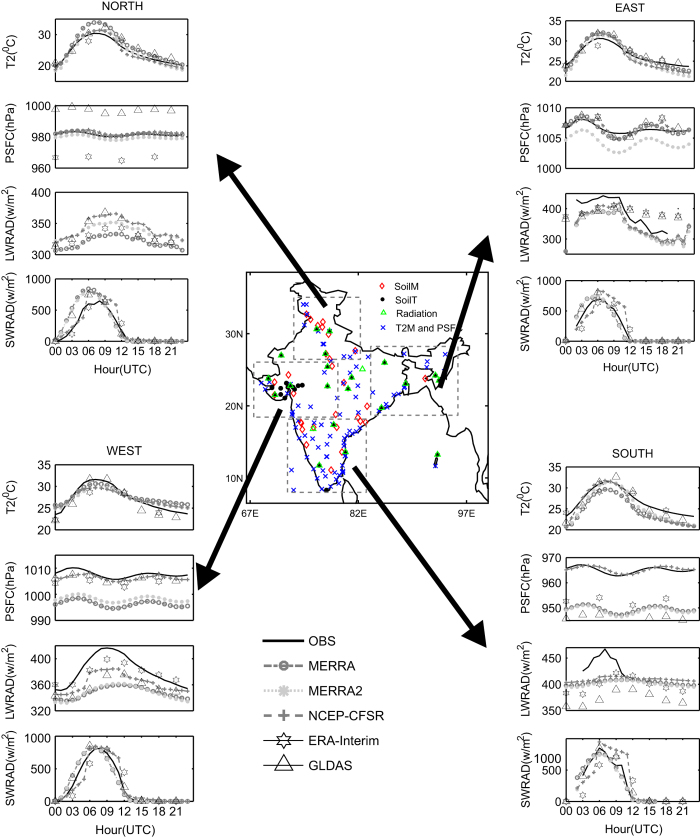
Observational station network used over the study domain and verification of diurnal variation of surface forcing from different reanalysis products at in-situ stations observations. (**a**) the diurnal cycle of temperature at 2 m (°C), surface pressure (hPa), surface downward short wave (W m^−2^), and long wave (W m^−2^) radiation obtained from MERRA, MERRA2, NCEP-CFSR, ERA-Interim, GLDAS are verified against observations for Northern parts of India. (**b**), (**c**) and (**d**) are same as (a) but for east, west and southern parts of India respectively. Note that the triangle (Δ) represents (22) Agromet stations for downward short wave and long wave observations and cross ( × ) mark represents (89) AWS station for air temperature and surface pressure observations. The solid black circle (•) represents soil temperature observations at 33 Agromet stations and red diamond represents 30 soil moisture observatories. The figure is generated using MATLAB 2015a, www.mathworks.com.

The near-surface atmospheric forcing parameters such as T2 and PSFC are compared with AWS observations from the India Meteorological Department (IMD) at 89 locations over the IMR for 2011–2013. The downward SWRAD and LWRAD at 22 Agro-Met locations and ST at 33 stations are obtained from the Meteorological and Oceanographic Satellite Data Archival Centre (MOSDAC) and as publicly available from http://www.mosdac.gov.in/. The SM observations at 30 sites are obtained from the Continental Tropical Convergence Zone (CTCZ) data bank (http://www.odis.incois.gov.in/). The validation of SM/ST is confined to the Indian summer monsoon months (June, July, August and September) for the period 2011–2013. The gridded (0.25° × 0.25°) satellite merged SM product (e.g., The global satellite-observed SM dataset (ESACCI^40^) developed by European Space Agency (ESA) under its Climate Change Initiative (CCI) program ; https://www.esa-soilmoisture-cci.org/) is used for study the SM variability. The network of observations used and their corresponding locations are shown in Fig. 1.

### Numerical experiments

A set of five integrations are conducted: Experiment1 (Ex1), Experiment2 (Ex2), Experiment3 (Ex3), Experiment4 (Ex4) and Experiment5 (Ex5) using LDAS at 4 km grid space, augmenting with five different sources for the period 2009–2013. The LDAS is integrated over the Indian domain as shown in Fig. 1. The forcing fields are provided in 1-h interval with an integration timestep of 15 min. Additional details of different reanalysis datasets used for these experiments are presented in Table 2.

The Advanced Wide Field Sensor^25^ (AWiFS) 56-meter 19-category landuse obtained from the National Remote Sensing Centre (NRSC: http://bhuvan.nrsc.gov.in/), green vegetation from 30 s resolution USGS green vegetation fraction^27^, and 1 km Food and Agriculture Organization of the United Nations/U.S. General Soil Map (FAO/STATSGO2) soil texture classifications^26^ are used. In all the five integrations, the static fields and rainfall forcing are maintained identical while other atmospheric forcing parameters are varied (Table 2). GLDAS radiation was selected as forcing due to its better agreement with observations and consistency (presented in the results section) following tests with other fields.

Indian summer monsoon rainfall accounts ~75% of country’s annual total rainfall. Monsoon depressions (MDs), off-shore troughs and meso-convective systems (MCSs) contribute significantly to total seasonal rainfall^41^. To demonstrate the credibility of the data in numerical models for simulation of heavy rainfall events, two heavy rainfall events (12 July and 14 August of 2010) over Delhi and one typical MD case (18–21 June 2011) affecting the east coast of India are identified during the monsoon season.

The Advanced Research version of the Weather Research Forecasting (ARW^28^) model (Version 3.7.1) is used to simulate these heavy rainfall events over the IMR. The same domain configuration as for the LDAS is adopted. In the model, there are 51 terrain following hydrostatic vertical pressure (sigma) coordinate placed close together in lower atmosphere (12 levels below 850 hPa and 22 levels below 500 hPa) and relatively coarser in the upper atmosphere. The grid structure follows Arakawa C grid. The Mellor-Yamada-Janjic (Eta) scheme for planetary boundary layer, Monin-Obukhov (Janjic Eta) scheme for surface physics, four layers Noah LSM for land surface, RRTM and Dhudhia schemes for longwave and shortwave radiation physics are used. WSM 6 microphysical parameterization is used with explicit convection. This model configuration is typically used for simulation of monsoon heavy rain events and is guided by prior studies.

A set of two numerical experiments were conducted. The CNTL run which uses climatological SM as surface conditions along with NCEP- FiNaL (FNL) analysis as initial and lateral boundary condition. In the second experiment (known as LDAS), the LDAS-based SM/ST are updated in place of climatological SM/ST product. This approach is typically adopted over IMR for rainfall prediction studies^10^. Except for the incorporation of initial SM/ST fields, there are no other differences in both experiments in terms of model configuration and physics.

### Code availability

The High Resolution Land Data Assimilation System (HRLDAS v3.4.1) is used to develop the dataset. The source code used is available through the National Center for Atmospheric Research (NCAR) at http://www.ral.ucar.edu/research/land/technology/hrldas.php. The WRF-ARW (Version 3.7.1) source code is available at NCAR as open source model (http://www2.mmm.ucar.edu/wrf/users/). These software have been compiled and run on Linux servers. Licensed Fortran and C compilers and other open source libraries, e.g., NetCDF and Jasper are required. More details are available at https://ral.ucar.edu/ and WRF users page (http://www2.mmm.ucar.edu/wrf/users/).

## Data Records

The SM/ST dataset in NetCDF format are freely available for download from *figshare* (Data Citation 1). The dataset at 3 hourly temporal frequencies are available for Indian summer monsoon season for the period 2001–2014. The SM/ST product can be used for various hydro-meteorological research applications.

Naming convention:

India.LDAS.SFC.SMST.yyyymmddhh.nc

variable: SOIL_M = volumetric soil moisture at 0-10 cm layer [m^3^ m^−3^]

SOIL_T = Soil temperature at 0-10 cm layer [K]

## Technical Validation

### Validation of atmospheric forcing

The near-surface atmospheric parameters such as T2, PSFC, SWRAD, and LWRAD are evaluated at all AWS and Agro-Met stations (Fig. 1). The hourly observational datasets collected from various sources are passed through quality control checks to ensure the integrity of the data from unrealistic and spurious values. Earlier studies have also performed similar quality checks for validation of NLDAS forcing^42–45^.

**Figure 2:**
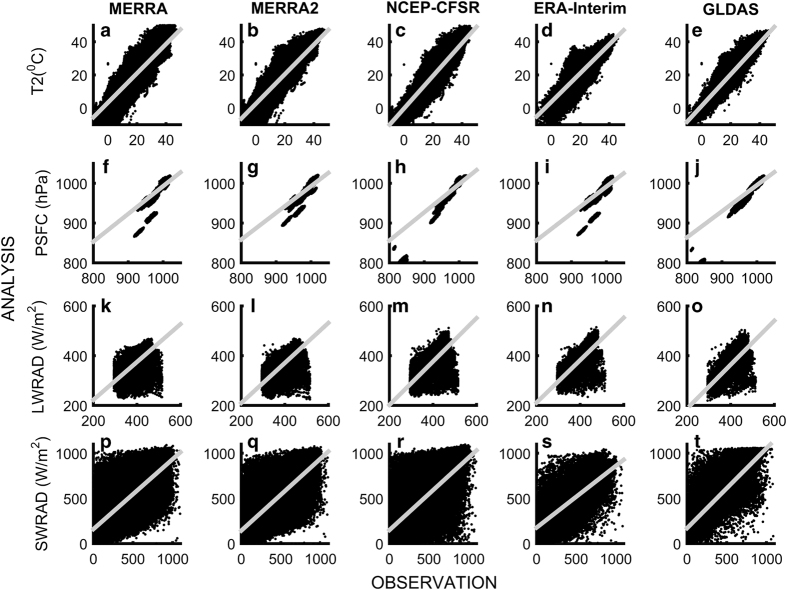
Verification of surface forcing parameters from different reanalysis products at in-situ station observations (shown in Fig. 1) for the period 2011–2013. Top row represents scatter diagram of temperature at 2 m (°C) obtained from (**a**) MERRA, (**b**) MERRA2, (**c**) NCEP-CFSR, (**d**) ERA-Interim, and (**e**) GLDAS. Solid gray lines show linear regression fits to the data. (**f**-**j**), (**k**-**o**) and (**p**-**t**) are same as (a-e) but for surface pressure (hPa), surface downward long wave (W m^−2^) and short wave (W m^−2^) radiation respectively. The figure is generated using MATLAB 2015a, www.mathworks.com.

The scatter diagrams of T2, PSFC, SWRAD, and LWRAD obtained from MERRA, MERRA2, CFSR, ERA-Interim, GLDAS, and observations for all the stations in conjunction with linear regression fit line (*y = ax ± b*) are shown in Fig. 2. Here, *a*≠1 indicates a model deviation and the index of model bias is b. The results show that the T2 (a = 0.98) and PSFC (a = 0.78) from CFSR performed relatively better followed by GLDAS. The parameter values, root mean square error (RMSE), and correlation coefficient (r) of T2, PSFC, downward solar radiation, and thermal radiation from all sources are presented in Table 3. The estimated correlations are statistically significant at the 95% level. These statistics are computed taking all station measurements into account for the period 2011–2013. The result shows that the CFSR analyses exhibited higher correlation of 0.91 for both T2 and PSFC, respectively, while MERRA, Era-Interim for T2 (r = 0.87) and Era-Interim for PSFC (r = 0.78) showed the least correlation. Similarly, the RMSE in T2 (2.85 °C) of GLDAS followed by CFSR (2.94 °C) had the lowest RMSE and PSFC (23.78 hPa) of CFSR had the lowest RMSE, while MERRA2, MERRA, and ERA exhibited the higher RMSE. The SWRAD and LWRAD from GLDAS showed a higher correlation coefficient ( > 0.75) with better agreement (a = 0.88 for both SWRAD and LWRAD) than the other products. Overall results indicate that the radiation products from GLDAS are better in comparison with other products.

**Figure 3:**
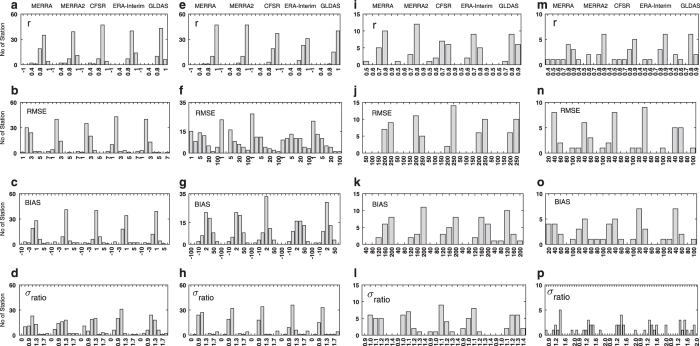
Comparison of error statistics of the surface forcing parameters from different reanalysis products at in-situ station observations (shown in Fig. 1) for the period 2011–2013. The first column represents number of stations per bin of (**a**) correlation (r), (**b**) root mean square error (RMSE), (**c**) mean bias (BIAS) and (**d**) the ratio of the standard deviation of the reanalysis product σ to that from the observations σ_obs_ (σ_ratio_), for temperature at 2 m (°C). (**e**-**h**), (**i**-**l**) and (**m**-**p**) are same as (a-d), but for surface pressure (hPa), surface downward short wave (W m^−2^), and long wave (W m^−2^) radiation respectively. The figure is generated using MATLAB 2015a, www.mathworks.com.

The histogram (showed in Fig. 3) illustrates the correlation, bias, ratio of standard deviations, and root-mean-square error for T2, PSFC, SWRAD and LWRAD. All of the data products have a correlation greater than 90% at most of the stations for T2. An exception was with MERRA products which shows ~20 station having correlation less than 90%. The RMSE for all of the products is within a range 0–3°C and the bias is in range -2–2 °C at most of the stations. The RMSE in the ERA Interim is the least among the group considered. The ratio of standard deviations also shows that all the products could capture the observed variation reasonably (ratio follows ~1) in T2 and ERA-Interim is found have more no of stations follow observed variability. Analyses of PSFC indicated that the correlation is greater than 90% for all the datasets. However, the error metrics indicated that CFSR is better than other products with less RMSE and bias. While the ERA interim found to have higher RMSE and bias. The ratio of standard deviations also shows that all the products represents the observed PSFC variation. The shortwave radiation is better in GLDAS with high correlation, least bias as compared to others. The ratio of standard deviations shows that MERRA and MERRA2 are superior in capturing observed SWRAD variation. There are no notable difference for the statistics of LWRAD among the data products.

The diurnal variation of the T2, PSFC, downward solar and thermal radiation from all sources at four different geographical locations (representative of north, east, west, and southern parts) of India is illustrated in Fig. 1. There are differences in range and shape of the diurnal cycle among the datasets for all parameters. In general, the shape of the observed diurnal cycle of T2 (r~0.97) and PSFC (r~0.80) is better reproduced in CFSR at all four geographical locations, however during 15–24 UTC T2 is underestimated in the eastern and southern locations in CFSR. The ERA-Interim temperature is underestimated at 06 UTC and close to observation at 12–18 UTC at all four locations. The mean diurnal T2 are better agreement in ERA-Interim and CFSR with observations (Supplementary Table S1) at all the location. While the correlation is better in CFSR, MERRA and MERRA2 and variations (standard deviation) are close to observations in ERA-interim. The mean diurnal PSFC are better agreement in CFSR while the correlation is better in MERRA and MERRA2. The LWRAD in all the datasets is underestimated at all locations (observations are not present in the north). The shape of the observed diurnal cycle of LWRAD is reasonably reproduced in CFSR for the east and west sub-domains but shows poor agreement in the south. The SWRAD from MERRA, MERRA2, and GLDAS reasonably follows the shape of the observed diurnal cycle at all four locations. However, CFSR SWRAD displayed a peculiar feature in representing the shape of the diurnal cycle. There are discontinuities from 05 UTC to 06 UTC and 11 UTC to 12 UTC SWRAD, i.e., the temporal gradient between the 5th hour forecast and successive hour reanalysis is high and are not consistent with observations. Similar discontinuities are found for the hourly CFSR temperature possibly due to the use of 6 hourly assimilation^46^.

Based on the above results, the T2 from CFSR, ERA and GLDAS show better skill with minimum RMSE and has the highest correlation coefficient in representing diurnal variation, while the SWRAD and LWRAD from GLDAS displayed better skill than the other products. The PSFC from CFSR is found to be better among the data products.

### Validation of land surface dataset

The LDAS is integrated for 2009 to 2013 using the above-mentioned reanalysis products as forcing (see Table 2). There are six SM/ST products, including GLDAS, that are validated against in-situ measurements (shown in Fig. 1) for 2011–2013. The SM/ST observations for monsoon months (JJAS) are obtained from the CTCZ data bank and are used for the validation.

**Figure 4:**
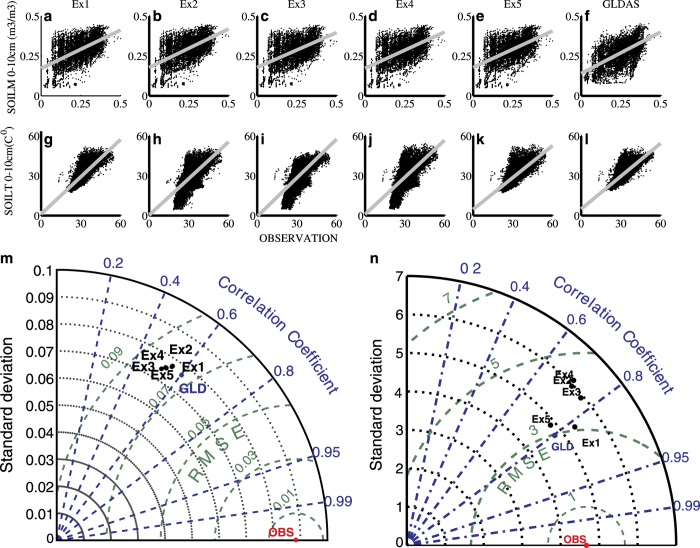
Verification of top layer (0-10 cm) soil moisture (m m^−3^) and soil temperature (°C) from various experiments of LDAS and GLDAS with in-situ observations (shown in Fig. 1) during monsoon season of 2011–2013. Top row represents scatter diagram of soil moisture from (**a**) Ex1, (**b**) Ex2, (**c**) Ex3, (**d**) Ex4, (**e**) Ex5, and (**f**) GLDAS against with in-situ observations. The second row (**g**-**l**) are same as (**a**-**f**) but for soil temperature. (**m**) represents mean statistics including standard deviation, root mean square error, and correlation coefficient of Ex1, Ex2, Ex3, Ex4, Ex5, GLDAS (GLD) through Taylor diagram for soil moisture (m m^−3^) and (**n**) is same as (**m**) but for soil temperature. The figure is generated using MATLAB 2015a, www.mathworks.com.

Fig. 4 shows the scatter plot (Fig. 4a–l) and Taylor diagram (Fig. 4m–n) of the surface layer (0–10 cm) SM/ST from five runs (Ex1, Ex2, Ex3, Ex4, and Ex5) along with GLDAS products against in-situ observations for the period 2011–2013. Solid gray lines represent the linear regression fit (*y = ax ± b*). The Taylor diagram illustrates standard deviation, root mean square error, and correlation coefficient of SM and ST. Figure 4g-l shows that the ST from Ex1, Ex5, and GLDAS are in better agreement with observations. The Taylor diagram of SM shows that GLDAS and Ex1 exhibited the least RMSE (0.07 m^3^ m^−3^) and higher correlation (0.60 for GLDAS and 0.55 for Ex1) followed by Ex2. The SM standard deviation of Ex1 (0.077 m^3^ m^−3^) and Ex2 (0.077 m^3^ m^−3^) is close to observations (0.088 m^3^ m^−3^) as compared to others. Table 4 shows that GLDAS SM has better skill followed by Ex1. The ST from Ex1 is closer to observations with higher correlation (r = 0.82) than other products. The RMSE of ST is least in both GLDAS and Ex1 (~3.00 °C) when compared to that of others. However, ST parameter values (a = 0.93, b = 1.18) are better for Ex1 than GLDAS (a = 0.82, b = 5.06).

The time series of daily surface layer SM/ST at four different locations with varying latitude is analyzed using six SM/ST (Ex1, Ex2, Ex3, Ex4, Ex5, and GLDAS) datasets for the monsoon period during 2011 (Supplementary Figure S1 & S2). The result shows that both the SM and ST fields could capture observed temporal variability across the six datasets and the SM/ST derived from Ex1 is in overall better agreement with observations than the others (including GLDAS SM/ST).

**Figure 5:**
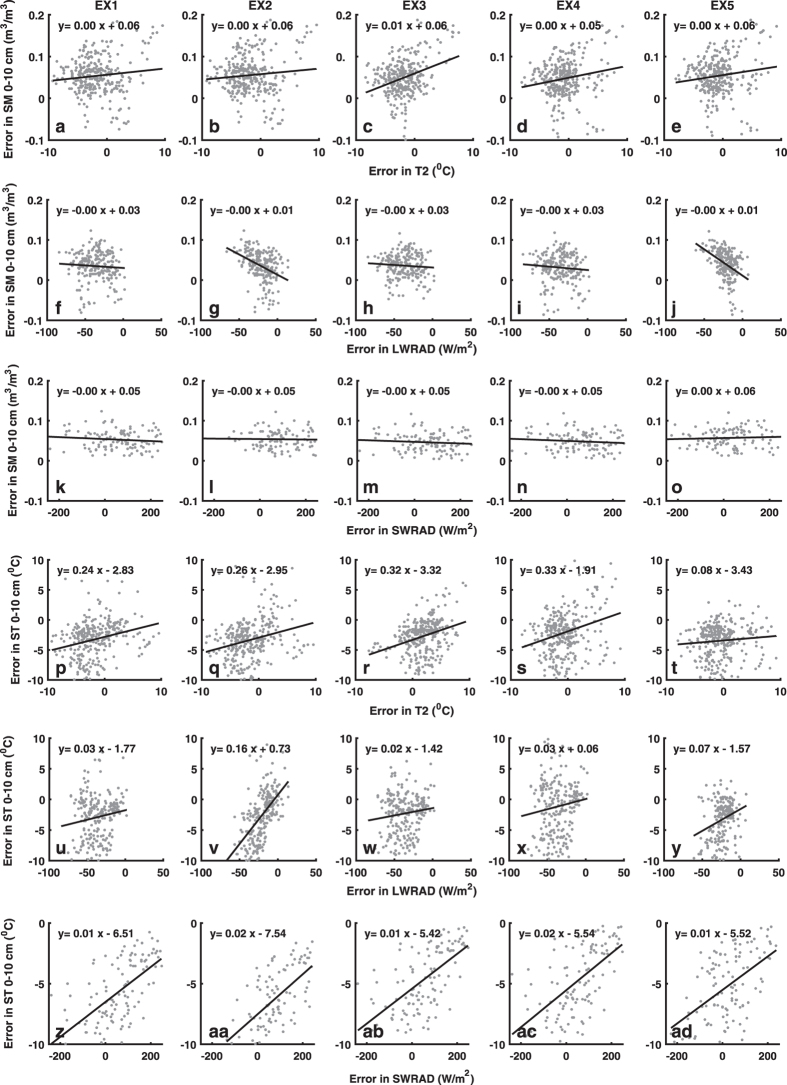
Relation between forcing error and LDAS output error. The top row presents scatter diagram of LDAS soil moisture (m^3^ m^−3^) error with error in temperature at 2 m (°C) obtained from **a**) Ex1, **b**) Ex2, **c**) Ex3, **d**) Ex4, **e**) Ex5. (**f**–**j**) and (**k**–**o**) are same as (**a**–**e**), but for surface downward long wave (W m^−2^), and short wave (W m^−2^) radiation respectively. (**p**-**ad**) is same as (**a**–**o**) but for LDAS soil temperature (°C). All the relationship is calculated at 95% confidence level. The figure is generated using MATLAB 2015a, www.mathworks.com.

Figure 5 shows the scatter diagram of error in SM/ST from all the experiments against error in T2, LWRAD and SWRAD. The intent of the analysis is to understand the relation between quality of forcing with the associated LDAS SM/ST. There is notable positive relation between error in ST and error in T2, LWRAD and SWRAD in all the experiments. This shows that the ST is sensitive to T2, LWRAD and SWRAD and error in these forcing parameter could lead to error in ST. Figure 5 shows no notable relation (trend ~0) between error in SM and error in T2 in all the experiment. This indicates SM is relatively less sensitive to T2. However, the relation between SM and radiation (the SWRAD and LWRAD) is complex and nonlinear.

### Utility of LDAS-based SMST data products

From the above results and discussion, it is inferred that the forcing fields used in Ex1 show consistently better agreement with available observations. Therefore, the LDAS is integrated for 2000–2014 using Ex1 configuration and results from the first year (2000) is considered for spin-up^47^ of the LDAS. The mean diurnal, seasonal and inter-annual variation of the LDAS is studied. Further, the utility of the SMST data is accessed through land initialization in meso-scale simulation of monsoon depression and heavy rain events. This land surface dataset obtained from the Ex1 configuration is referred to as the IMR NMM-SMST LDAS dataset and is available for download. The NMM stands for National Monsoon Mission, which is the flagship project under which these efforts were undertaken.

### SMST variability during monsoon season:

The diurnal cycle of LDAS ST (°C) at four different locations (78.00°E, 30.33°N; 77.45°E, 27.20°N; 70.44°E, 21.50°N; and 78.59°E, 17.34°N) across India for the summer monsoon season as whole, as well as dry and wet days of monsoon 2011 is analyzed (Supplementary Figure S3). It is noted that the LDAS ST follows the observed diurnal cycle for all locations except in the north (78.00°E, 30.33°N). However, the amplitude of the simulated diurnal cycle is higher than observed and is greater for ‘dry’ days than ‘wet’ days. This is likely a signature of the heat thermal coefficient which is a complex function of soil texture^48^ (and quartz content).

The time series of daily surface layer SM/ST over four different regions (north, east, west and south as shown in Fig. 1) is analyzed (Supplementary Figure S4 & S5) for the monsoon period during 2011. The result shows that both the SM and ST could capture the observed temporal variability for all the four regions. However, SM also shows a positive bias for the four regions. This is likely due to the uncertainty in TRMM rainfall inputs^39^ and the higher soil field capacity and soil field porosity parameters used in the LDAS system. The ST agrees well with observations across all the regions with the exception of south.

**Figure 6:**
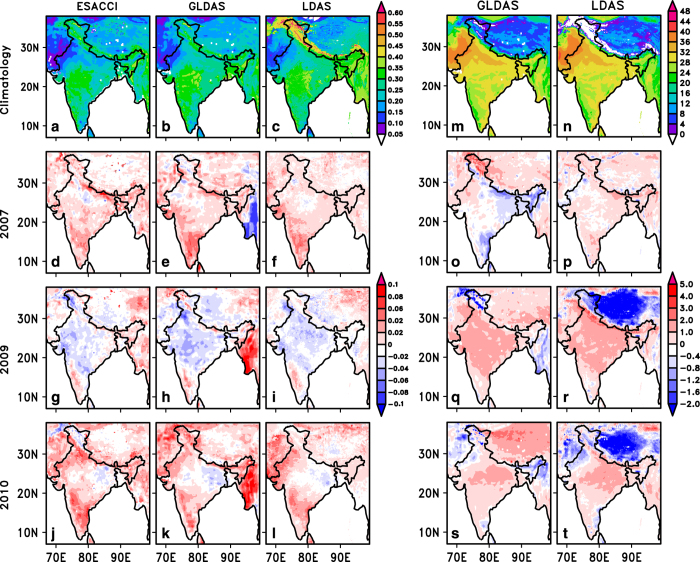
Spatial distribution of soil moisture (m^3^ m^−3^) and soil temperature (°C) climatology and their anomalies in contrasting monsoon years. The JJAS climatology from (**a**) satellite (ESACCI), (**b**) GLDAS and (**c**) LDAS are presented. (**d**–**f**), (**g**–**i**) and (**j**–**l**) are same as (a–c) but for soil moisture anomaly for the year 2007 (an above normal monsoon year), 2009 (a deficit year) and 2010 (normal year) respectively. The JJAS soil temperature climatology from (**m**) GLDAS and (**n**) LDAS are presented. (**o**–**p**), (**q**–**r**) and (**s**–**t**) are same as (**m**–**n**), but for soil temperature anomaly for the year 2007, 2009 and 2010. The figures are generated using open source software GrADS (Grid Analysis and Display System) version 2.1.0 (http://cola.gmu.edu/ grads/downloads.php).

Figure 6a-l illustrates the spatial distribution of SM climatology (2001–2014) during monsoon season from ESACCI^40^ satellite merged product, GLDAS, and the SM/ST dataset (LDAS) developed in this study. Seasonal anomaly of SM for different monsoon seasons such as i) above normal monsoon year (2007), ii) deficit monsoon year (2009), and iii) normal monsoon year (2010) are reviewed. The Indian monsoon rainfall for 2007, 2009, and 2010 was 108%, 82%, and 99% of Long Period Average (LPA) respectively (http://www.tropmet.res.in). It may be noted that India did not experience an above normal monsoon during the last two decades (http://www.tropmet.res.in), thus 2007 monsoon year, which was close to the above normal category ( > 110% of LPA), is treated as an above normal monsoon year in the present study.

The spatial distribution of LDAS SM shows good variability in comparison with GLDAS and ESACCI, however, LDAS exhibited wet bias in the Himalaya. This led to relatively less spatial correlation in LDAS SM (0.73) than GLDAS (0.93) when verified with the ESACCI SM. This has been verified by calculating the correlation excluding the Himalayan region and found that it increases to 0.81. This lower correlation was reviewed further and found to be primarily due to the Himalayan ice/snow region where the quality/reliability of LDAS forcing is questionable. Preliminary analysis revealed that the land use obtained from NRSC (http://bhuvan.nrsc.gov.in) has higher land ice spatial extent over Himalaya. Another possibility could be the rainfall uncertainties in this region^38^. Further, the higher correlation of GLDAS could be due to similar grid spacing of ESACCI. In spite of this wet bias in the Himalayas, such high spatial and temporal resolution (4 km and 3-hrly) data is useful for studying variability at different (sub-daily, monthly and seasonal as well as sub-divisional) scales. Interestingly, the temporal correlation of gridded SM during monsoon season of 2001-2014 clearly exhibited the higher correlation for LDAS than GLDAS (Supplementary Figure S6). The Fig. 6a-c shows that the magnitude of SM over west central India is higher (the range is 0.35-0.4m^3^ m^−3^) and least over west north-west India^49^ (the range is 0.05-0.15 m^3^ m^−3^) from all the data products and is consistent with the rainfall distribution over the IMR. This result is supported by a previous study as well^50^. It is noteworthy that the central monsoon region is identified as an SM coupling hot spot and is dominated by clayey soil. The clay soil has hydraulic conductivity and allows greater moisture retention for longer time periods as compared to sandy soil, for example. The LDAS could demarcate higher SM over the Himalayan ice region and this is likely due to the representation of Indian land-use where the snow and ice classification are well defined. The SM anomaly from LDAS shows better match than GLDAS with the ESACCI reference dataset for above normal year (2007) and normal year (2010) and deficit year (2009). The anomaly also has a better match over south peninsular India, northern Jammu and Kashmir region for the above normal year (2007) and normal year (2010) and central India region during deficit year (2009). However, GLDAS shown superiority over the northeast regions during 2007 and 2010. The pattern correlation of SM anomaly has shown improvement in LDAS (r = 0.35, r = 0.50 r = 0.51) over GLDAS (r = 0.17, r = 0.47, r = 0.35) for the 2007, 2009, 2010 periods.

Figure 6m-t shows the spatial distribution of ST climatology from GLDAS, LDAS along with seasonal anomaly of ST during 2007, 2009 and 2010. Since satellite ST is not available, only a comparison between GLDAS and LDAS is presented. The spatial distribution of ST climatology follows GLDAS over Indian region except Himalayan region where the ST is below freezing (<0) while in GLDAS it is much warmer. There are positive anomalies (in the range 1-2 °C) found in both LDAS and GLDAS ST during deficit year (2009) and normal year (2010). The GLDAS ST shows negative anomaly during above normal year 2007 and no notable anomaly was seen in the developed SM/ST fields. For the normal year, soil is typically wet and cooler while in the deficit year, soil is relatively dry and hence warm. Thus, the LDAS data product could capture the inter-annual variability in accordance to the variation in monsoon rainfall, which is consistent with the known hydro-climatology of the region^51^.

The spatial distribution of SM/ST with progression of the Indian summer monsoon is analyzed by studying SM/ST conditions during June, July, August, and September months. The results show (Supplementary Figure S7) that the land surface condition (SM/ST) has coherence with rainfall distribution and progress of the Indian monsoon rainfall. The SM reaches saturation in July over most parts of the IMR as monsoon rainfall covers the Indian region with central India having higher soil moisture (~0.4 m^3^ m^−3^) in July, August, and September.

The inter-annual variation of monthly SM/ST derived from LDAS and GLDAS is studied for contrasting months such as April, August, November as representative of summer, monsoon, and autumn season, respectively (Supplementary Figure S8 & S9). There are distinct inter-annual variations of SM for each of the three representative months both in the LDAS and GLDAS, and the variations differ on a regional and seasonal basis. The inter-annual variation of SM in August follows the variability of Indian monsoon rainfall^52^ i.e., contrasting SM variations with higher variability in the northwest and lower in northeast India. The high resolution LDAS SM has shown superiority over GLDAS in capturing inter-annual variation over northeast India and the Himalayan belt.

### Application to weather forecast

**Figure 7:**
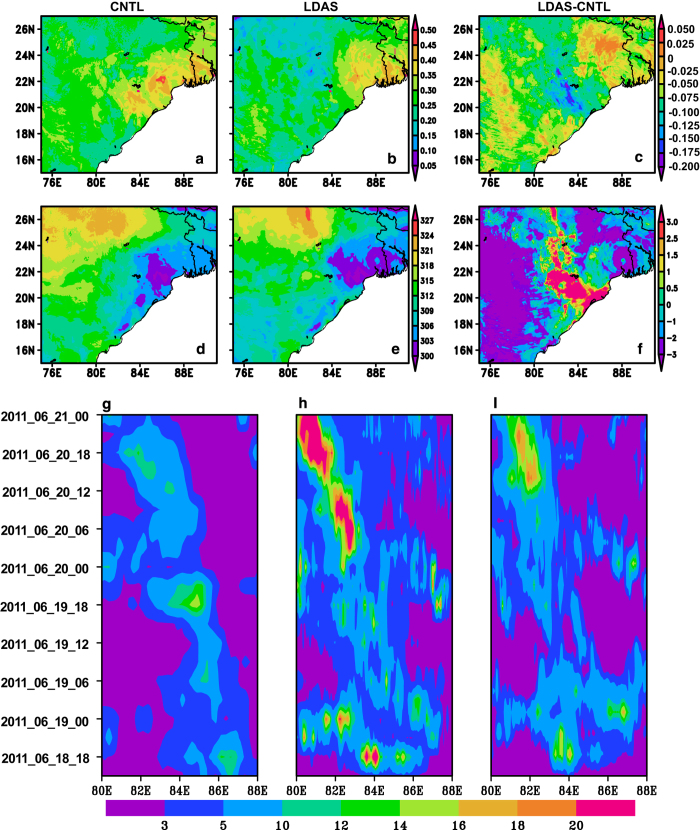
Impact of LDAS soil moisture (m^3^ m^−3^) and soil temperature (°C) on model initialization and subsequent rainfall simulation associated with monsoon depression. Spatial distribution of top layer (0-10 cm) soil moisture (m^3^ m^−3^) from (**a**) CNTL, (**b**) LDAS and (**c**) LDAS- CNTL valid for initial time 12 UTC, 18 June 2011. (**d**–**f**) are same as (**a**–**c**) but for soil temperature (K). The time-longitude crossection of 3 hourly rainrate (mm hr^−1^) rate obtained from (**g**) TRMM, (**h**) CNTL and (i) LDAS are presented. The figures are generated using open source software GrADS (Grid Analysis and Display System) version 2.1.0 (http://cola.gmu.edu/grads/downloads.php).

In this section, the impact of SM/ST fields in simulating of one monsoon depression (MD) and two heavy rainfall cases are presented. Figure 7 presents initial 0–10 cm SM and ST from both CNTL and LDAS and their difference (LDAS-CNTL) for comparison, for a typical MD case valid at 12 UTC 18 June 2011. There are notable difference between the LDAS-SM (Fig. 7b) and CNTL-SM (Fig. 7a). The initial SM and ST condition is much more heterogeneous in LDAS than CNTL experiment. The LDAS-ST is also warmer by 2–3.5°C (Fig. 7f) than CNTL over western part of the study region. However, LDAS-ST over the depression region (21–25 °N, 84–88 °E) is cooler by 0–1.5°C. The initial land condition is relatively wet and cool in LDAS than CNTL (21–25 °N, 84–88 °E).

The time-longitude cross-section of 3-hrly rain rate obtained from CNTL and LDAS runs and the corresponding TRMM rainfall is shown in Fig. 7g-i. This rainfall is averaged over a 23 °N – 25 °N latitudinal box. It is clear that there is overestimation in the 3 hourly rain rate for the CNTL run compared to that of observed as well as the LDAS-based model run. That is, the rainfall amount is better simulated in the LDAS-based model simulation. The effect of land surface heterogeneity in providing the forcing for reproducing realistic precipitation amounts and distribution is demonstrated in previous studies^53^. Prior results have led to the understanding that the land surface heterogeneity influences the lower atmosphere and gradually extends the impact to the middle layers. The relatively warmer land condition in the depression region (21–25 °N, 84–88 °E) owing to high sensible heat flux in the CNTL run. This could trigger severe convection leading to overestimation of rainfall in the CNTL. On the other hand, the LDAS experiment results in realistic rainfall due to less sensible heat flux from relatively cooler land condition in the depression region. The sensible heat flux should be sufficiently available for the initiation of severe convection along with latent heat flux (higher SM content)^54^.

**Figure 8:**
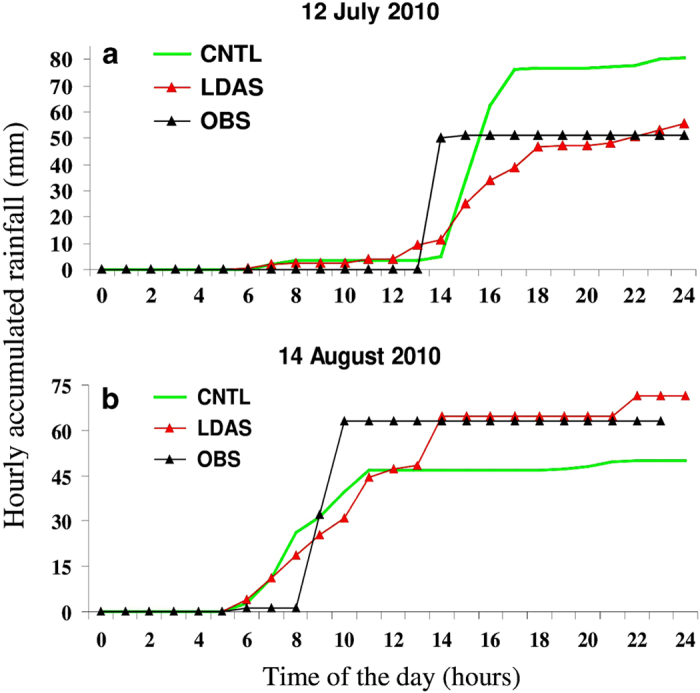
Impact of LDAS soil moisture (m^3^ m^−3^) and soil temperature (°C) initialization on subsequent rainfall simulation of severe convection over New Delhi, India. Verification of hourly accumulated rainfall (mm) obtained from CNTL, LDAS with rain gauge observations (OBS) for (**a**) 12 July 2010 and (**b**) 14 August 2010 convective cases. The figure is generated using Microsoft office 2013, www.microsoft.com.

Taking another example of heavy rainfall, Fig. 8 shows hourly accumulated rainfall distribution at a station in New Delhi (28.7^°^ N, 77.1^°^E) corresponding to thunderstorm cases that occurred on 12 July 2010 and 14 August 2010. There is a peak rainfall of 4 cm and 6.5 cm that is observed at 14 UTC 12 July 2010 and 10 UTC 14 August 2010, respectively. The simulation results again clearly highlight the superiority of the LDAS (red solid line) experiment where the high-resolution SM/ST fields developed in this study, are employed as initial conditions. For the 12 July 2010 case, both the CNTL and LDAS have lead-time rainfall at 6 UTC and the CNTL rainfall has increased to 8 cm at 15 UTC. On the other hand, LDAS simulation could capture the observed peak rainfall magnitude relatively well. In case of the 14 August 2010 heavy rainfall event, the CNTL run shows its deficiency (~2 cm) in predicting observed rainfall (~6.5 cm) where as LDAS run could accurately capture the observed rainfall amount. Similar experiments were conducted for a number of different cases (not shown) and these results consistently highlight the superiority of LDAS-based simulations. That is, from the process-scale perspective the realistic representation of land surface and SM/ST heterogeneity played key role in improving the rainfall prediction^10^. These experiments clearly demonstrate the credibility and utility of the developed SM/ST dataset for improving coupled weather models.

In general, when the 4 km data are aggregated to a coarser grid corresponding to the resolution used in the GLDAS, the land surface characteristics between LDAS and GLDAS are at par for SM, but the LDAS fields remain superior for ST. The LDAS captured the observed diurnal variation of ST. The intra-seasonal and inter-annual variation of SM and ST are also delineated clearly in the high-resolution dataset. This dataset is available to the user community for the monsoon period from 2001 to 2014 under the National Monsoon Mission.

## Additional Information

**How to cite this article**: Nayak, H. P. *et al*. High-resolution gridded soil moisture and soil temperature datasets for the Indian monsoon region. *Sci. Data*. 5:180264 doi: 10.1038/sdata.2018.264 (2018).

**Publisher’s note**: Springer Nature remains neutral with regard to jurisdictional claims in published maps and institutional affiliations.

## Supplementary Material



Supplementary information

Supplementary Table

## Figures and Tables

**Table 1:** Reanalysis/forecast products used for validation of T2 (°C), PSFC (hPa), SWRAD (W m^−2^), and LWRAD (W m^−2^).

Source	Spatial resolution (Lat° x Lon°)	Temporal resolution(Hour)
MERRA	0.5000 × 0.6667	Hourly
MERRA2	0.5000 × 0.6250	Hourly
ERA- INTERIM	0.75 × 0.75LWRAD, SWRAD (0.25 × 0.25)	6 Hourly(3 Hourly)
NCEP-CFSR	0.204 × 0.204	Hourly
GLDAS	0.2500 × 0.2500	3 Hourly

**Table 2:** Data used in developing the SM/ST fields using the LDAS.

Parameter	Sources	Interpolation
Land use	19-category 30 s resolution NRSC land use	No interpolation from WRF grid to LDAS grid
vegetation	30 s resolution USGS green vegetation fraction	No interpolation from WRF grid to LDAS grid
Soil texture	16-category 5-min USGS soil texture	No interpolation from WRF grid to LDAS grid
Precipitation	TRMM hourly precipitation at 0.25^°^resolution	Mass conservation interpolation
**Experiment-1**		
Atmospheric forcing	CFSR hourly data at (0.204^°^ X 0.204^°^) resolution (T2, specific humidity, PSFC, U and V wind)	Bilinear interpolation
Radiation	GLDAS 3 hourly SWRAD and LWRAD radiation at 0.25^°^resolution	Linear interpolation to hourly and bilinear in space
**Experiment-2**		
Atmospheric forcing	CFSR hourly data at (0.204^°^ X 0.204^°^)(T2, specific humidity, PSFC, U and V wind, SWRAD and LWRAD)	Bilinear interpolation
**Experiment-3**		
Atmospheric forcing	ERA-Interim hourly data at (0.75^°^ × 0.75^°^) resolution (T2, specific humidity, PSFC,U and V wind)	Bilinear interpolation
Radiation	GLDAS 3 hourly SWRAD and LWRAD at 0.25^°^resolution	Linear interpolation to hourly and bilinear in space
**Experiment-4**		
Atmospheric forcing	MERRA hourly data at (1/2^°^ × 2/3^°^) resolution (T2, specific humidity, PSFC, U and V wind)	Bilinear interpolation
Radiation	GLDAS 3 hourly SWRAD and LWRAD at 0.25^°^resolution	Linear interpolation to hourly and bilinear in space
**Experiment-5**		
Atmospheric forcing	MERRA hourly data at (1/2^°^ × 2/3^°^) resolution (T2, specific humidity, PSFC, U and V wind, SWRAD and LWRAD)	Bilinear interpolation

**Table 3:** Parameter values for linear regression fits (*y = ax ± b*), correlation coefficient (r) and root mean square error (RMSE) for T2 (°C), PSFC (hPa), SWRAD (W m^−2^) and LWRAD (W m^−2^) obtained from each analysis/forecast products to in-situ observations.

Parameter	Product	a	b	r	RMSE
T2	MERRA	0.88	3.30	0.87	3.30
	MERRA2	0.89	2.58	0.89	3.09
	NCEP-CFSR	0.98	−0.30	0.91	2.94
	ERA-Interim	0.86	3.61	0.87	3.25
	GLDAS	0.95	1.28	0.91	2.85
PSFC	MERRA	0.76	233.58	0.79	36.91
	MERRA2	0.72	272.26	0.81	34.73
	NCEP-CFSR	0.78	217.59	0.91	23.78
	ERA-Interim	0.72	268.46	0.78	37.05
	GLDAS	0.74	250.97	0.90	26.44
SWRAD	MERRA	0.79	148.16	0.74	210.95
	MERRA2	0.79	142.75	0.76	202.70
	NCEP-CFSR	0.80	149.04	0.70	237.54
	ERA-Interim	0.70	163.95	0.70	212.59
	GLDAS	0.88	170.08	0.80	217.80
LWRAD	MERRA	0.76	70.17	0.70	41.72
	MERRA2	0.80	48.69	0.73	42.93
	NCEP-CFSR	0.87	27.25	0.75	39.04
	ERA-Interim	0.85	40.71	0.75	35.22
	GLDAS	0.88	10.10	0.75	48.13

**Table 4:** Parameter values for linear regression fits (*y = ax ± b*) for ST (°C), and SM (m m^−3^) obtained from Ex1, Ex2, Ex3, Ex4, Ex5, and GLDAS products to in-situ observations.

Parameter	Product	a	b
SM	Ex1	0.48	0.171
	Ex2	0.48	0.177
	Ex3	0.43	0.178
	Ex4	0.45	0.172
	Ex5	0.45	0.178
	GLDAS	0.52	0.14
ST	Ex1	0.93	1.18
	Ex2	0.96	−0.72
	Ex3	0.99	−1.57
	Ex4	0.97	−0.01
	Ex5	0.80	4.71
	GLDAS	0.82	5.06
