# Supplementary material for: High-resolution gridded soil moisture and soil temperature datasets for the Indian monsoon region
Source: Sci Data. 2018 Nov 20;5:180264. doi: 10.1038/sdata.2018.264 (PMC6244185; doi:10.1038/sdata.2018.264)

**List of Supplementary Figures**

**Figure S1:** Verification of soil moisture from five LDAS experiments and GLDAS using in-situ station observations. The daily time series of soil moisture (m^3^ m^-3^) at 0-10 cm depth obtained from Ex1, Ex2, Ex3, Ex4, Ex5, and GLDAS are compared against in-situ observations at (a-d) four different geographical locations in India during 2011 monsoon season (July– September). [3]

**Figure S2:** Verification of soil temperature from five LDAS experiments and GLDAS using in-situ station observations. The daily time series of soil temperature (^°^C) at 0-10 cm depth obtained from Ex1, Ex2, Ex3, Ex4, Ex5, and GLDAS are compared against in-situ observations at four different geographical locations in India during 2011 summer monsoon season (June – September). [4]

**Figure S3:** Verification of the soil temperature diurnal cycle for varying soil moisture conditions. The mean diurnal cycle of soil temperature (^°^C) at four different geographical locations across India corresponding to (left column) summer monsoon JJAS, (center) DRY, and (right column) WET days in 2011. The vertical bars are the standard deviation for each hour. DRY day is the mean diurnal cycle corresponding to no rain days and WET is the mean diurnal cycle during rainy days. [5]

**Figure S4:** Verification of soil moisture across different geographical regions in India. The daily time series of soil moisture (m^3^ m^-3^) at 0-10 cm depth of observed and LDAS for a) north, b) east, c) south, and d) west regions. [6]

**Figure S5:** Verification of soil temperature across different geographical regions in India. The daily time series of soil temperature (^°^C) at 0-10 cm depth of observed and LDAS for a) north, b) east, c) south, and d) west regions. [7]

**Figure S6:** Temporal correlation analysis of ESACCI soil moisture with GLDAS and LDAS. The temporal correlation of monsoon SM between (a) ESACCI and GLDAS for the period 2001-2014. (b) is same as (a) but for correlation between ESACCI and LDAS. The red color is confidence interval at 99%. The domain averaged correlation is 0.58 and 0.41 for LDAS and GLDAS respectively. [8]

**Figure S7:** The spatial distribution of soil moisture and soil temperature fields corresponding to the progress of Indian summer monsoon. The spatial distribution of average rainrate (mm/hr) for a) June, b) July, c) August and d) September months (monsoon period). e)-h) are the same as (a-d), but for soil moisture (m^3^/m^3^) at a 0-10 cm layer, i)-l) are the same as (a-d), but for mean soil temperature (^°^C) at the 0-10 cm layer. m)-p) are same as (e-h), but for 10-40 cm layer and q)-t) is same as (i-l), but for 10-40 cm layer. The average is computed using the data from 2001 to 2014. [9]

**Figure S8:** Inter-annual variation of soil moisture for contrasting months over India. The standard deviation of LDAS monthly soil moisture (m^3^/m^3^) at 0-10 cm layer for the months of a) April (APR), b) August (AUG), and c) November (NOV), and d) -f) is the same as a)-c), but for GLDAS. (g-l) are same as (a-f), but for 10-40 cm layer. [10]

**Figure S9:** Inter-annual variation of soil temperature for contrasting months over India. The standard deviation of LDAS monthly soil temperature (^°^C) at 0-10 cm layer for the months of a) April (APR), b) August (AUG), and c) November (NOV), and d) -f) are the same as a)-c), but for GLDAS. (g-l) are same as (a-f), but for 10-40 cm layer. [11]

**Supplementary figures**

**Figure S1:** Verification of soil moisture from five LDAS experiments and GLDAS using in-situ station observations. The daily time series of soil moisture (m^3^ m^-3^) at 0-10 cm depth obtained from Ex1, Ex2, Ex3, Ex4, Ex5, and GLDAS are compared against in-situ observations at (a-d) four different geographical locations in India during 2011 monsoon season (July– September)


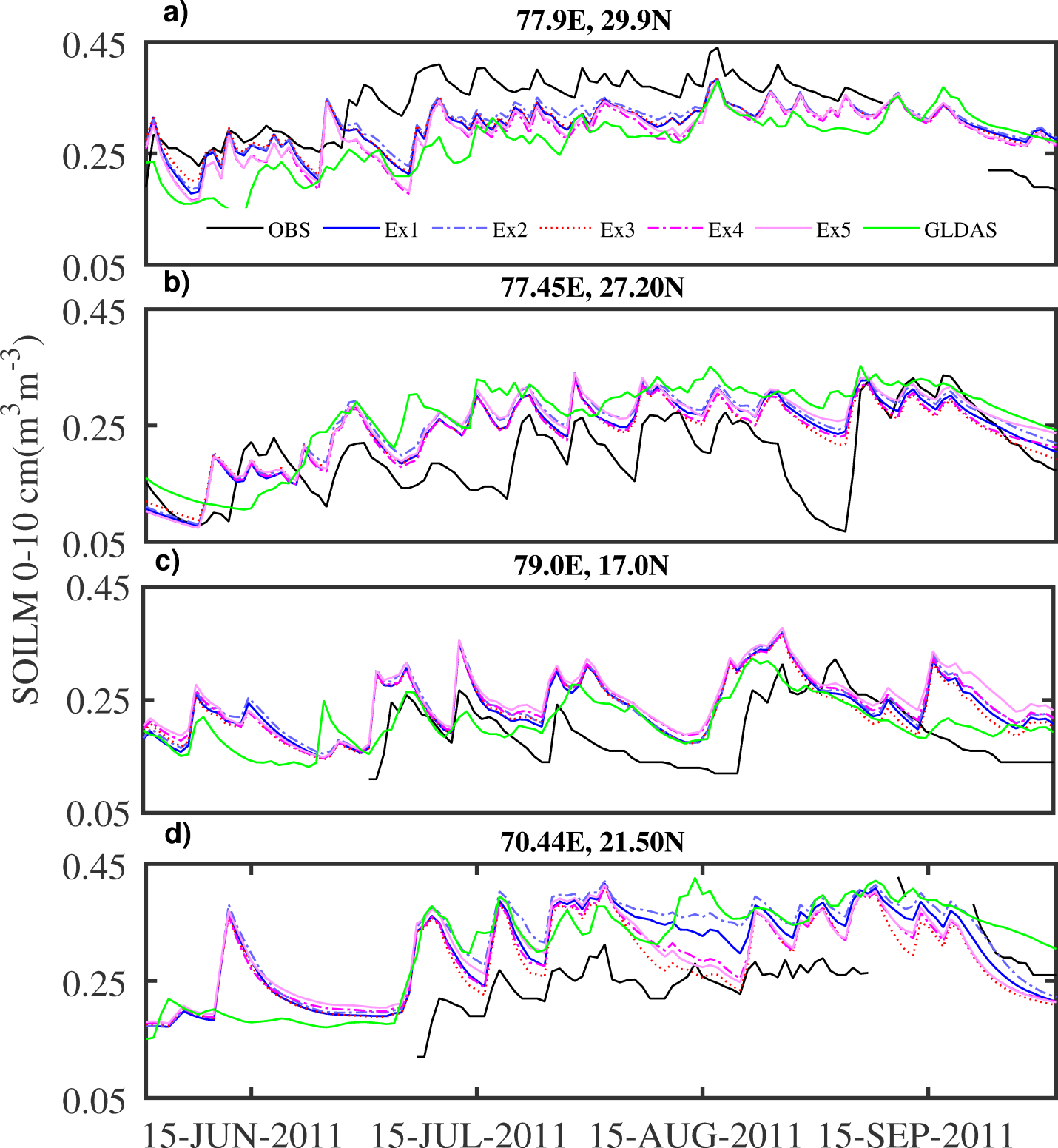

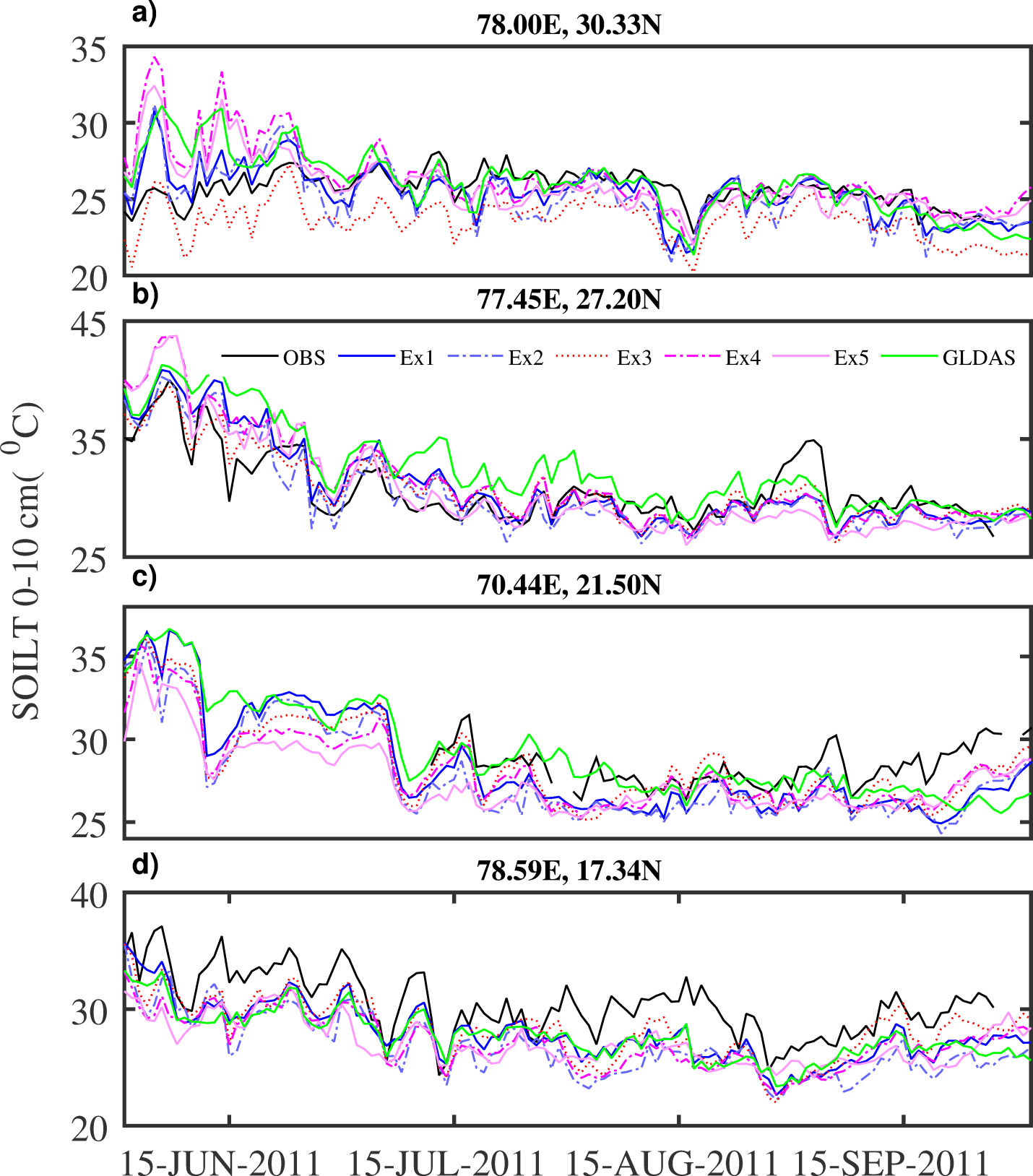


**Figure S2:** Verification of soil temperature from five LDAS experiments and GLDAS using in-situ station observations. The daily time series of soil temperature (^°^C) at 0-10 cm depth obtained from Ex1, Ex2, Ex3, Ex4, Ex5, and GLDAS are compared against in-situ observations at four different geographical locations in India during 2011 summer monsoon season (June – September).

**Figure S3:** Verification of the soil temperature diurnal cycle for varying soil moisture conditions. The mean diurnal cycle of soil temperature (^°^C) at four different geographical locations across India corresponding to (left column) summer monsoon JJAS, (center) DRY, and (right column) WET days in 2011. The vertical bars are the standard deviation for each hour. DRY day is the mean diurnal cycle corresponding to no rain days and WET is the mean diurnal cycle during rainy days.


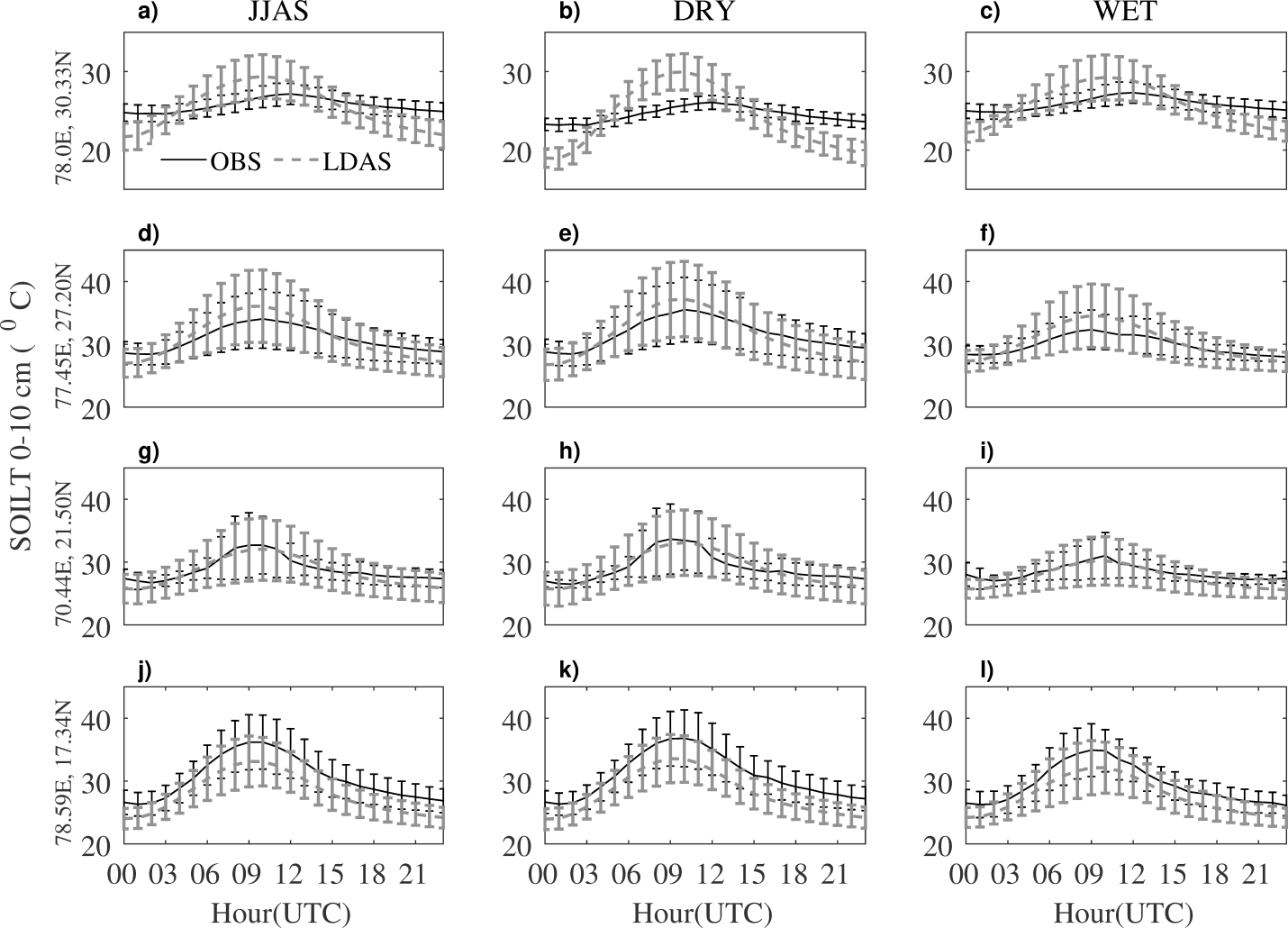


**Figure S4:** Verification of soil moisture across different geographical regions in India. The daily time series of soil moisture (m^3^ m^-3^) at 0-10 cm depth of observed and LDAS for a) north, b) east, c) south, and d) west regions.


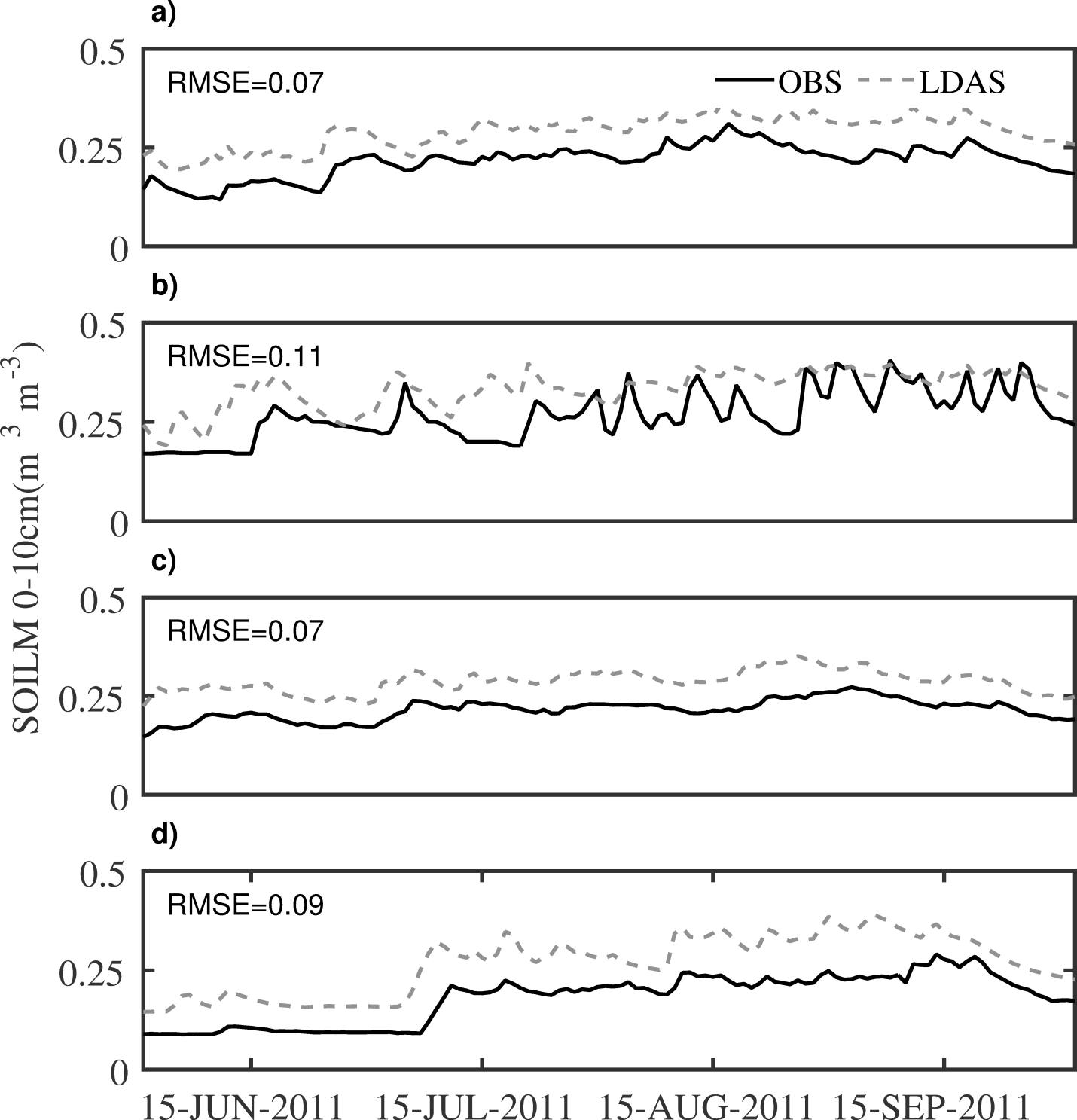


**Figure S5:** Verification of soil temperature across different geographical regions in India. The daily time series of soil temperature (^°^C) at 0-10 cm depth of observed and LDAS for a) north, b) east, c) south, and d) west regions.


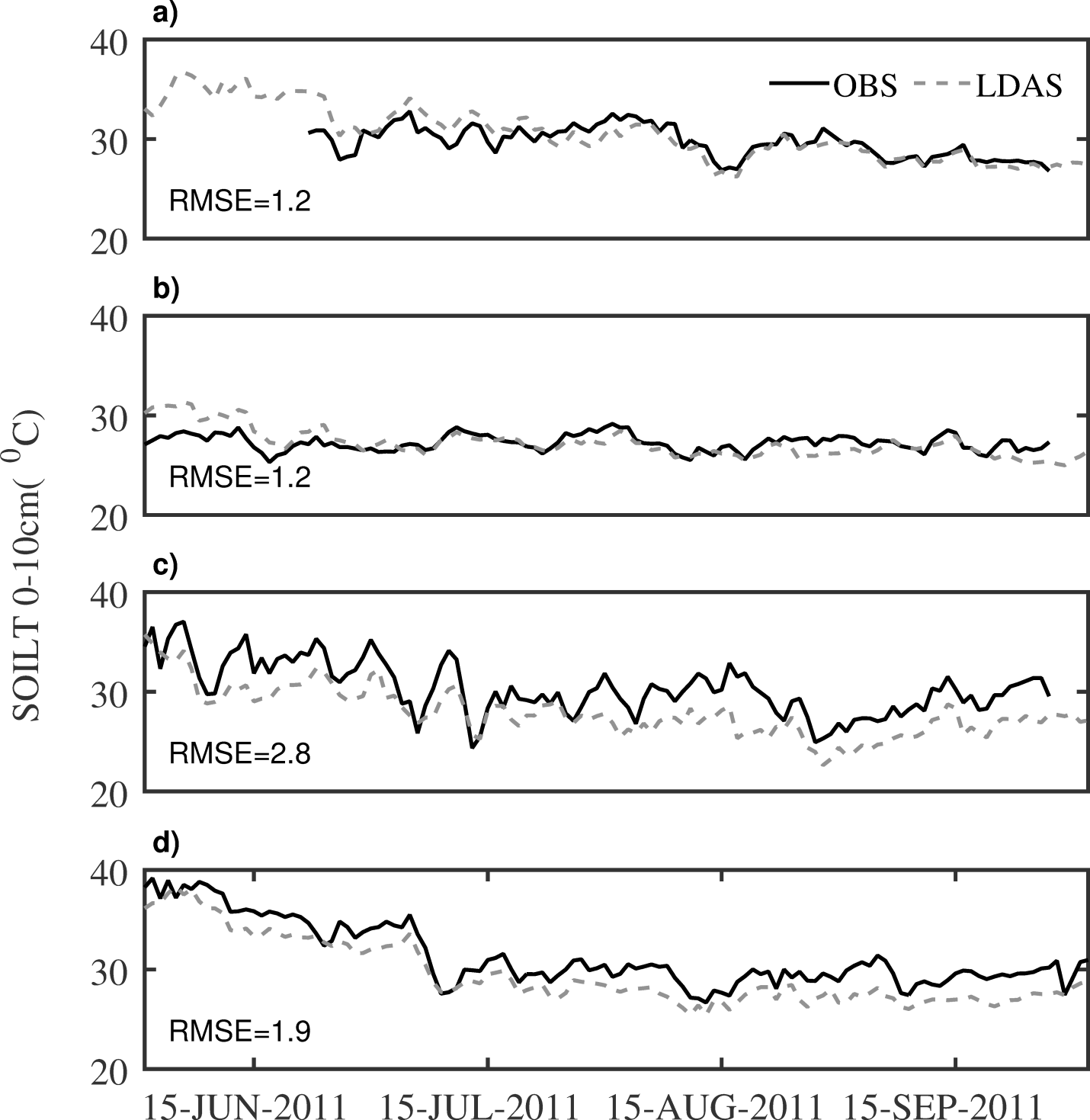

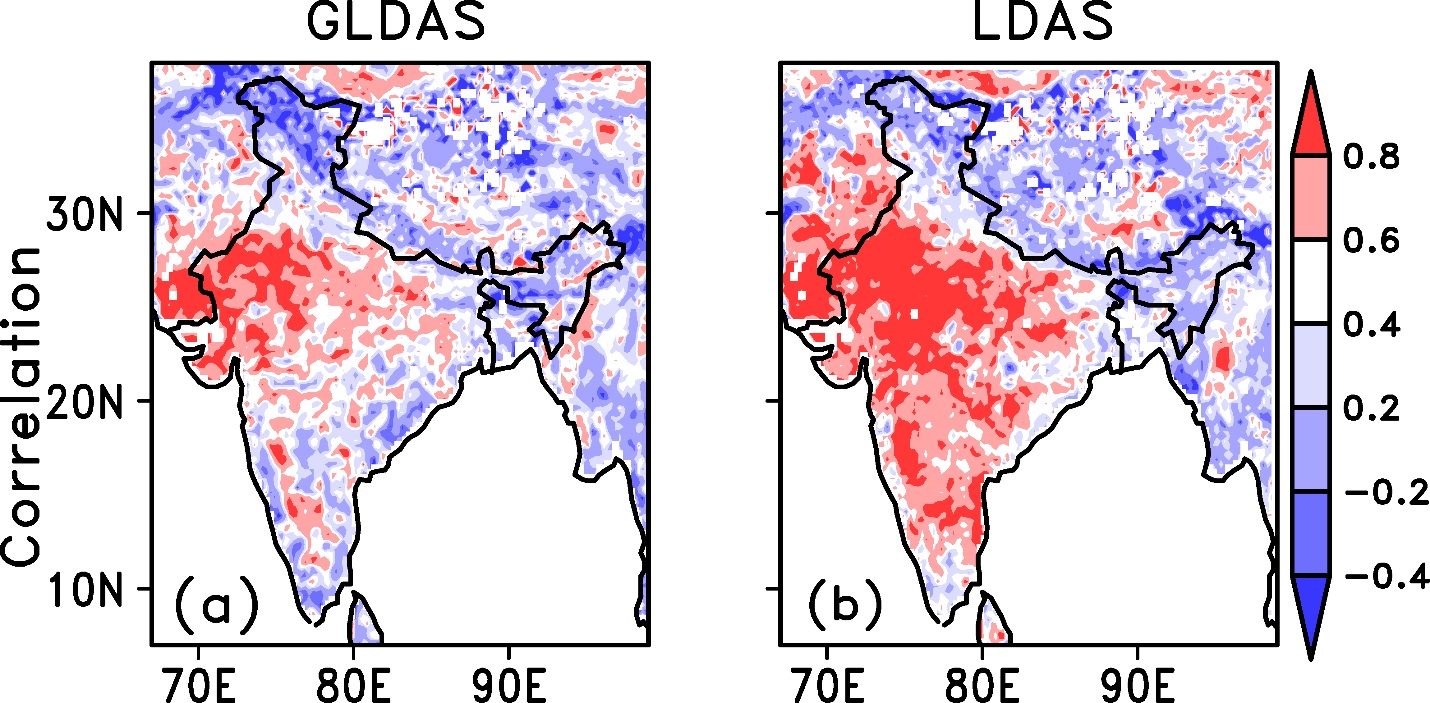


**Figure S6:** Temporal correlation analysis of ESACCI soil moisture with GLDAS and LDAS. The temporal correlation of monsoon SM between (a) ESACCI and GLDAS for the period 2001-2014. (b) is same as (a) but for correlation between ESACCI and LDAS. The red color is confidence interval at 99%. The domain averaged correlation is 0.58 and 0.41 for LDAS and GLDAS respectively.

**Figure S7:** The spatial distribution of soil moisture and soil temperature fields corresponding to the progress of Indian summer monsoon. The spatial distribution of average rainrate (mm/hr) for a) June, b) July, c) August and d) September months (monsoon period). e)-h) are the same as (a-d), but for soil moisture (m^3^/m^3^) at a 0-10 cm layer, i)-l) are the same as (a-d), but for mean soil temperature (^°^C) at the 0-10 cm layer. m)-p) are same as (e-h), but for 10-40 cm layer and q)-t) is same as (i-l), but for 10-40 cm layer. The average is computed using the data from 2001 to 2014.


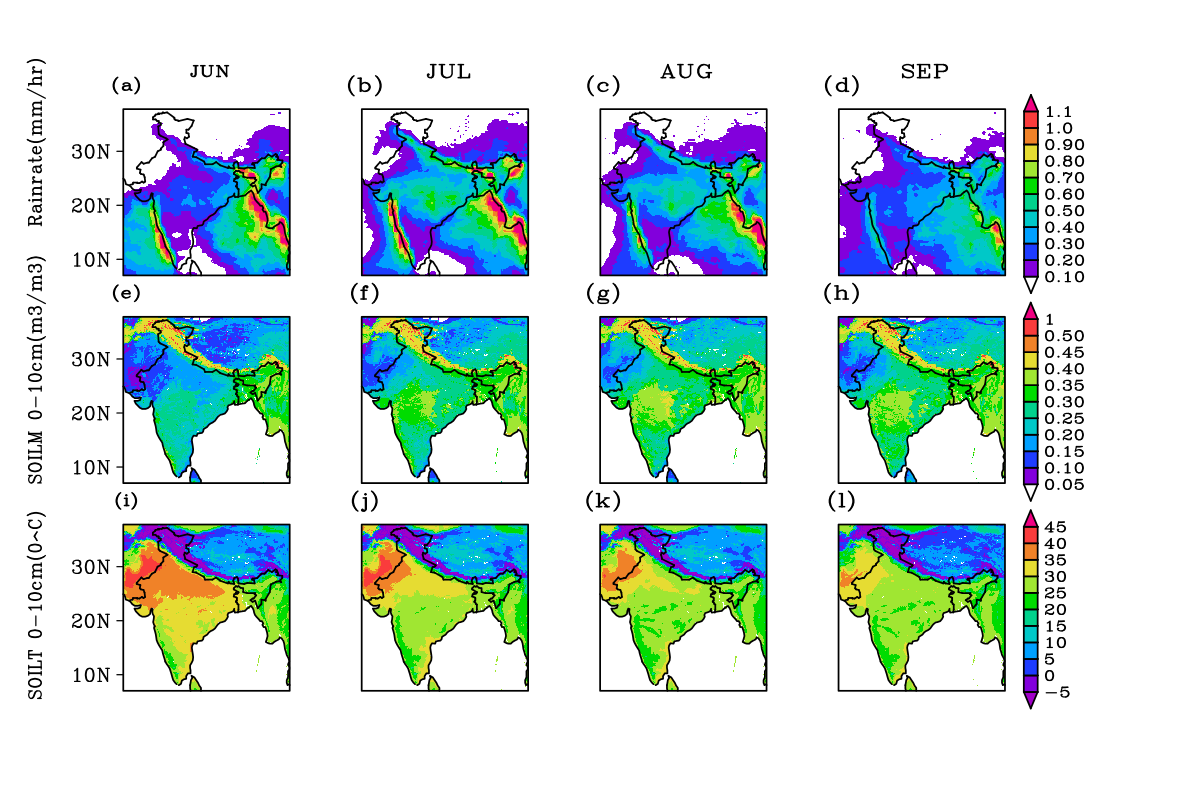

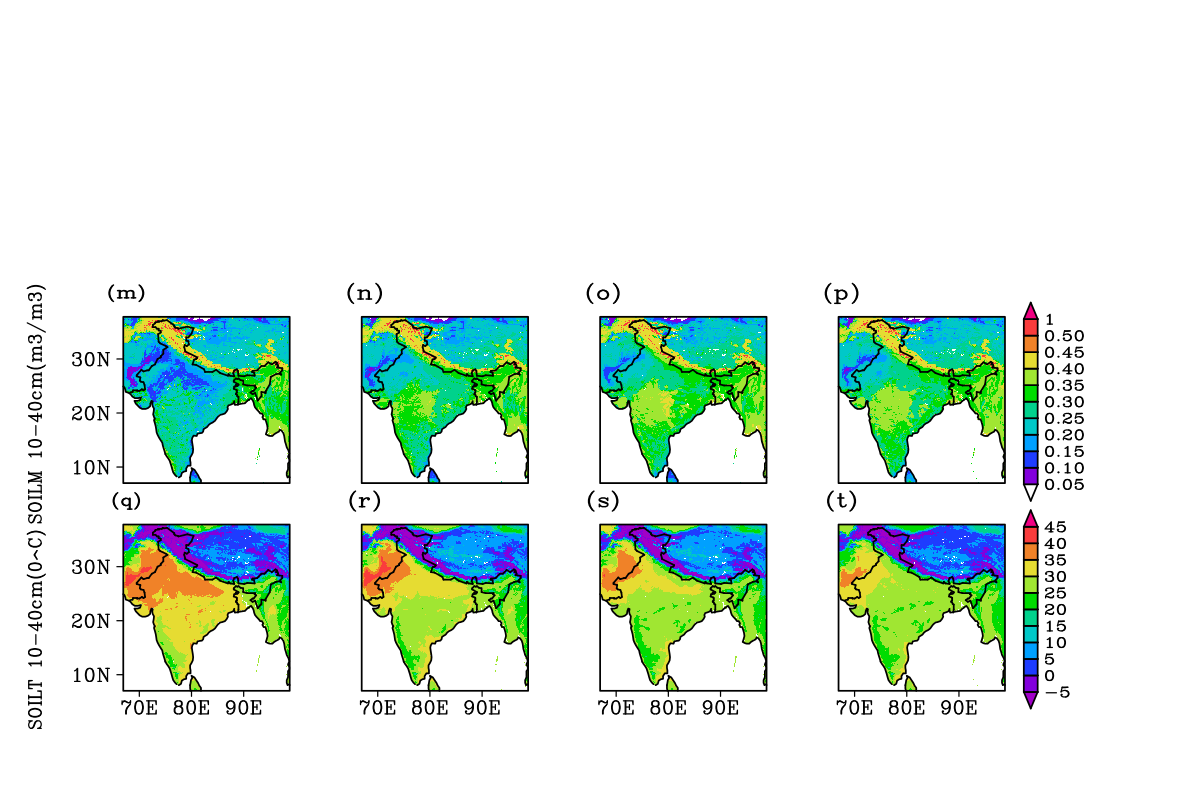


**Figure S8:** Inter-annual variation of soil moisture for contrasting months over India. The standard deviation of LDAS monthly soil moisture (m^3^/m^3^) at 0-10 cm layer for the months of a) April (APR), b) August (AUG), and c) November (NOV), and d) -f) is the same as a)-c), but for GLDAS. (g-l) are same as (a-f), but for 10-40 cm layer.


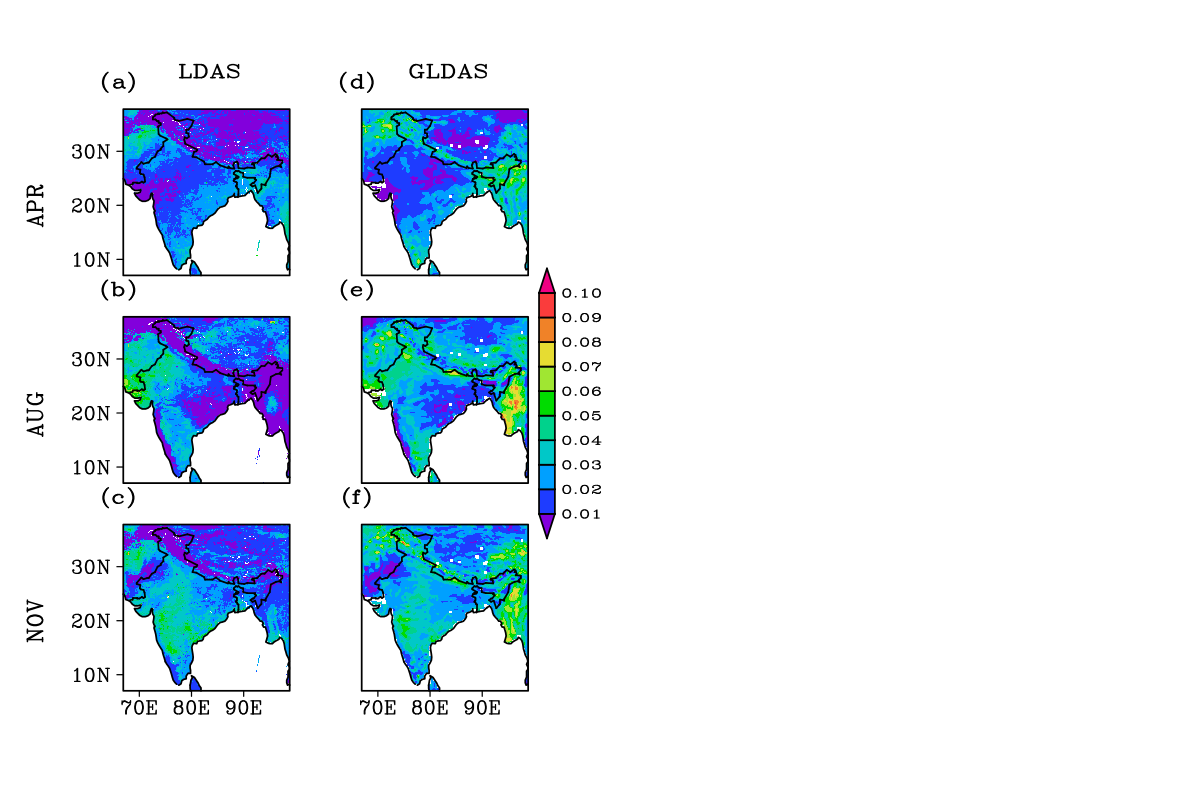

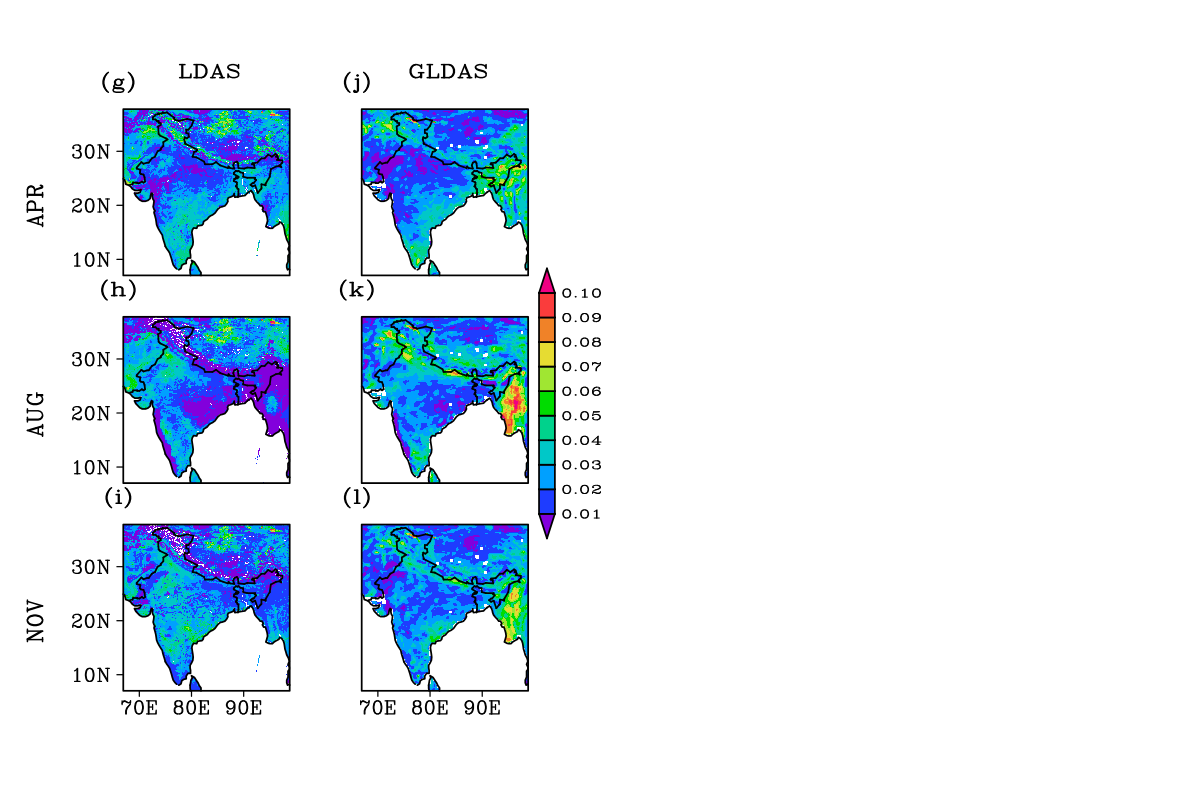


**Figure S9:** Inter-annual variation of soil temperature for contrasting months over India. The standard deviation of LDAS monthly soil temperature (^°^C) at 0-10 cm layer for the months of a) April (APR), b) August (AUG), and c) November (NOV), and d) -f) are the same as a)-c), but for GLDAS. (g-l) are same as (a-f), but for 10-40 cm layer.


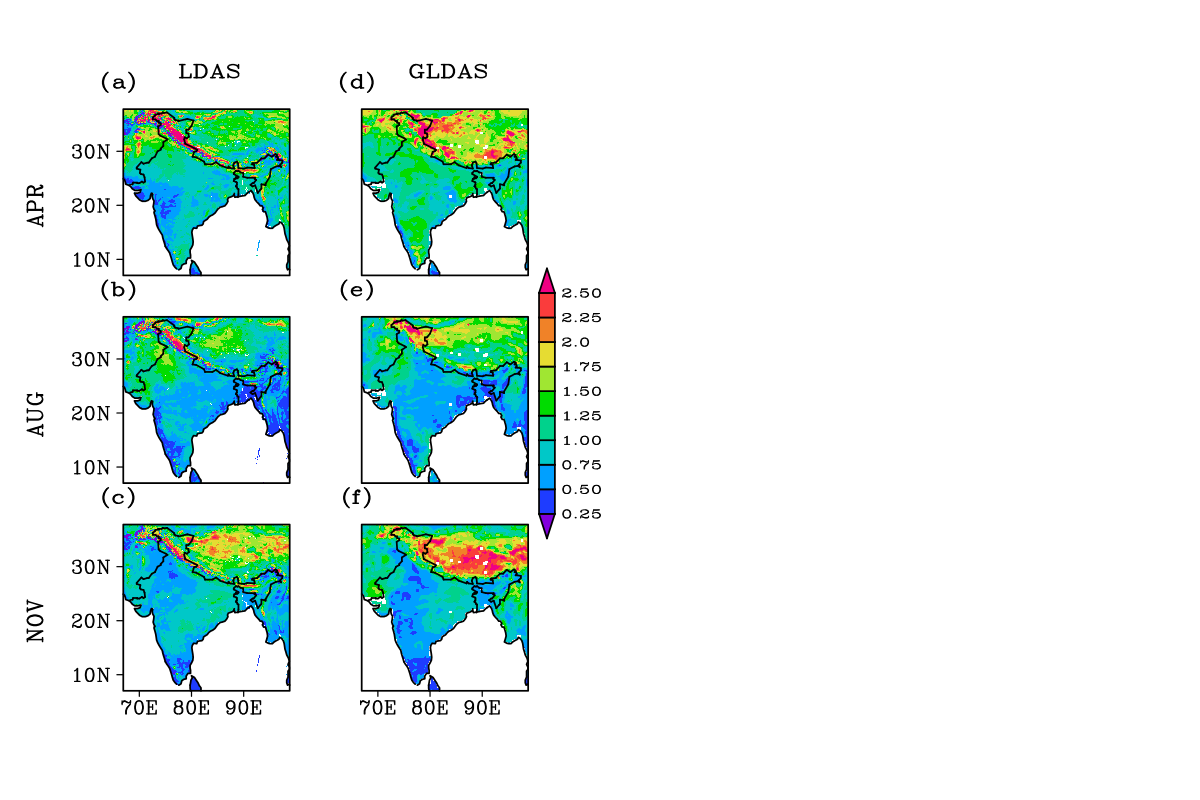

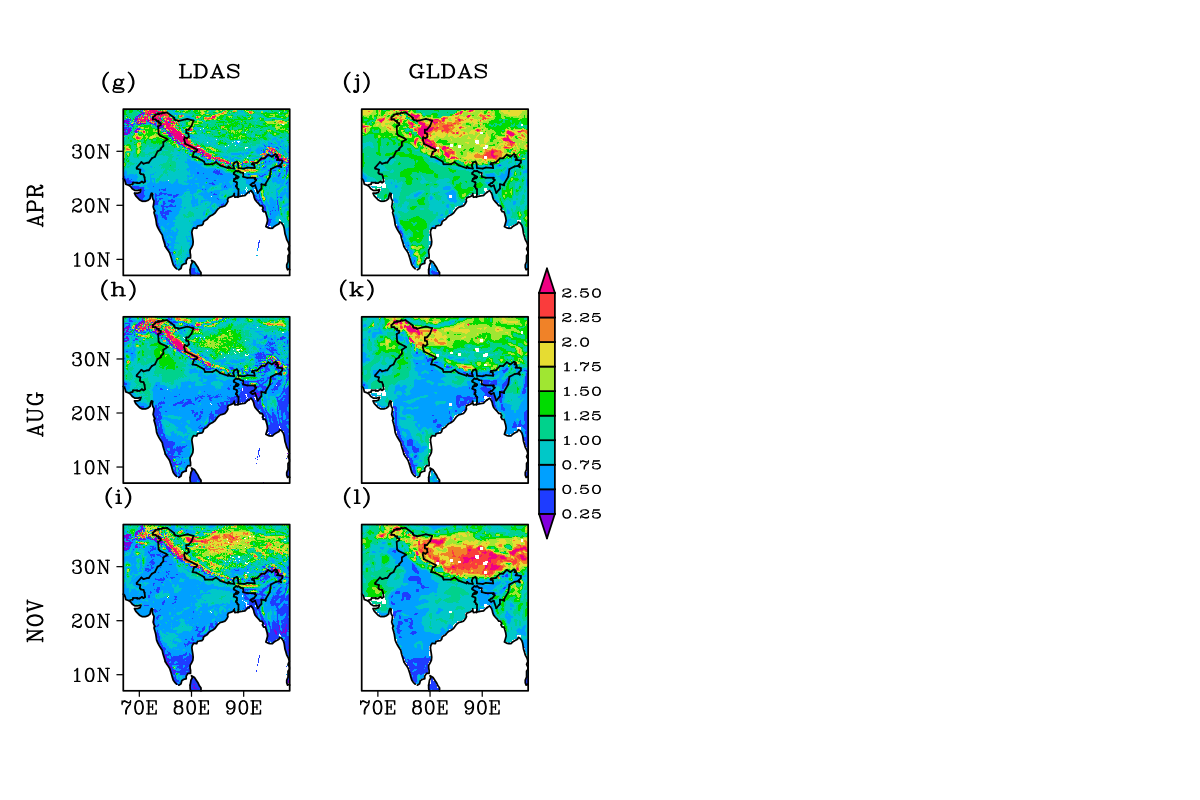

Supplement: Supplementary information [file sdata2018264-s2.docx]
