# Supplementary material for: High-resolution gridded soil moisture and soil temperature datasets for the Indian monsoon region
Source: Sci Data. 2018 Nov 20;5:180264. doi: 10.1038/sdata.2018.264 (PMC6244185; doi:10.1038/sdata.2018.264)
Supplement: Supplementary Table [file sdata2018264-s3.docx]

| **Source** | **OBS** | **MERRA** | **MERRA2** | **CFSR** | **ERA-Interim** | **GLDAS** |
| --- | --- | --- | --- | --- | --- | --- |
| T2(East) | | | | | | |
| **r** | - | 0.99 | 0.99 | 0.99 | 0.93 | 0.97 |
| **mean** | 26.56 | 26.80 | 25.73 | 26.20 | 26.40 | 27.25 |
| **std** | 2.45 | 3.37 | 3.60 | 3.54 | 2.22 | 3.01 |
| **T2(North)** | | | | | | |
| **r** | - | 0.99 | 0.99 | 0.99 | 0.91 | 0.95 |
| **mean** | 24.75 | 26.34 | 24.44 | 24.52 | 24.77 | 26.12 |
| **std** | 3.65 | 4.81 | 4.57 | 4.38 | 3.94 | 3.98 |
| **T2(South)** | | | | | | |
| **r** | - | 0.99 | 0.99 | 0.97 | 0.95 | 0.97 |
| **mean** | 26.77 | 24.57 | 25.47 | 25.35 | 26.60 | 26.34 |
| **std** | 2.94 | 3.13 | 3.75 | 4.05 | 2.64 | 4.17 |
| **T2(West)** | | | | | | |
| **r** | - | 0.98 | 0.98 | 0.99 | 0.93 | 0.98 |
| **mean** | 27.20 | 27.70 | 27.06 | 27.18 | 25.86 | 26.33 |
| **std** | 2.86 | 1.71 | 1.77 | 1.65 | 3.21 | 3.83 |
| **PSFC(East)** | | | | | | |
| **r** |  | 0.91 | .92 | 0.81 | 0.71 | 0.77 |
| **mean** | 1006.5 | 1006.6 | 1004.3 | 1006.7 | 1007.4 | 1007.1 |
| **sdt** | 0.71 | 1.11 | 1.08 | 1.01 | 1.33 | 1.36 |
| **PSFC(North)** | | | | | | |
| **r** |  | 0.98 | 0.97 | 0.80 | 0.93 | 0.95 |
| **mean** | 981.5 | 981.8 | 979.4 | 982.7 | 966.5 | 996.9 |
| **std** | 0.93 | 1.03 | 1.03 | 0.99 | 1.16 | 1.45 |
| **PSFC(South)** | | | | | | |
| **r** |  | 0.95 | 0.96 | 0.76 | 0.60 | 0.73 |
| **mean** | 965.0 | 949.4 | 948.8 | 965.3 | 952.8 | 945.8 |
| **std** | 1.28 | 1.22 | 1.34 | 1.13 | 1.33 | 1.16 |
| **PSFC(West)** | | | | | | |
| **r** |  | 0.94 | 0.90 | 0.77 | 0.91 | 0.92 |
| **mean** | 1007.7 | 996.3 | 998.2 | 1006.3 | 1004.3 | 1006.0 |
| **std** | 1.28 | 1.17 | 1.02 | 0.92 | 1.13 | 1.11 |

Supplementary Table S1

Table S1: The correlation (r), mean and standard deviation (std) for the diurnal cycle of T2 (^°^C) and PSFC (hPa), SWRAD (W m^-2^) and LWRAD (W m^-2^) at different locations (east, north, south and west) obtained from MERRA, MERRA2, NCEP-CFSR, ERA-Interim, GLDAS, and observations.

*continued...*

| **Source** | **OBS** | **MERRA** | **MERRA2** | **CFSR** | **ERA-Interim** | **GLDAS** |
| --- | --- | --- | --- | --- | --- | --- |
| **LWRAD(East)** | | | | | | |
| **r** | - | 0.90 | 0.91 | 0.91 | 0.47 | 0.41 |
| **mean** | 388.75 | 337.7 | 333.3 | 346.0 | 390.0 | 381.48 |
| **std** | 47.3 | 43.4 | 46.6 | 47.8 | 13.6 | 13.5 |
| **LWRAD (North)** | | | | | | |
| **r** | - | - | - | - | - | - |
| **mean** | - | 319.9 | 337.0 | 342.6 | 328.1 | 336.1 |
| **std** | - | 9.0 | 9.8 | 15.4 | 10.4 | 20.4 |
| **LWRAD (South)** | | | | | | |
| **r** | - | - | - | - | - | - |
| **mean** | - | 402.9 | 400.4 | 409.5 | 397.8 | 372.3 |
| **std** |  | 4.1 | 4.8 | 3.7 | 14.9 | 13.01 |
| **LWRAD (West)** | | | | | | |
| **r** |  | 0.88 | 0.93 | 0.95 | 0.96 | 0.86 |
| **mean** | 381.9 | 348.0 | 347.4 | 362.7 | 377.5 | 356.1 |
| **std** | 22.8 | 8.7 | 10.7 | 14.2 | 14.5 | 13.5 |
| **SWARD(East)** | | | | | | |
| **r** |  | 0.96 | 0.96 | 0.92 | 0.92 | 0.99 |
| **mean** | 216.2 | 223.3 | 226.4 | 248.7 | 206.6 | 240.2 |
| **std** | 275.2 | 296.4 | 302.6 | 321.0 | 273.0 | 330.1 |
| **SWARD (North)** | | | | | | |
| **r** |  | 0.92 | 0.92 | 0.96 | 0.97 | 0.96 |
| **mean** | 189.3 | 263.0 | 247.7 | 236.6 | 199.7 | 238.3 |
| **std** | 244.4 | 325.9 | 306.3 | 300.0 | 262.9 | 311.8 |
| **SWARD (South)** | | | | | | |
| **r** |  | 0.97 | 0.98 | 0.86 | 0.68 | 0.97 |
| **mean** | 383.8 | 374.4 | 348.1 | 436.8 | 215.7 | 264.0 |
| **std** | 330.6 | 308.6 | 300.2 | 370.0 | 288.5 | 350.7 |
| **SWARD (West)** | | | | | | |
| **r** |  | 0.96 | 0.96 | 0.96 | 0.90 | 0.99 |
| **mean** | 238.6 | 247.0 | 249.5 | 246.8 | 240.6 | 275.7 |
| **std** | 310.8 | 317.5 | 320.1 | 329.2 | 317.0 | 358.4 |
